# How Often Does an Individual Trial Agree with Its Corresponding Meta-Analysis? A Meta-Epidemiologic Study

**DOI:** 10.1371/journal.pone.0113994

**Published:** 2014-12-04

**Authors:** Wilson W. S. Tam, Jin-Ling Tang, Meng-yang Di, Kelvin K. F. Tsoi

**Affiliations:** 1 Alice Lee Centre for Nursing Studies, Yong Loo Lin School of Medicine, National University of Singapore, Kent Ridge, Singapore, Singapore; 2 Jockey Club School of Public Health and Primary Care, Faculty of Medicine, The Chinese University of Hong Kong, Shatin, Hong Kong; 3 The Hong Kong Branch of the Chinese Cochrane Centre, Faculty of Medicine, The Chinese University of Hong Kong, Shatin, Hong Kong; 4 Shenzhen Key Laboratory for Health Risk Analysis, Shenzhen Research Institute of the Chinese University of Hong Kong, Shenzhen, China; 5 Big Data Decision Analytics Research Centre, The Chinese University of Hong Kong, Shatin, Hong Kong; Bambino Gesù Children's Hospital, Italy

## Abstract

**Objective:**

A meta-analysis may provide more conclusive results than a single trial. The major cost of meta-analysis is the time of waiting before the meta-analysis becomes available and resources spent on consequent trials that may not be necessary. The objective of this study is to address how often the result of a single trial, in particular the first trial, differs from that of its corresponding meta-analysis so as to reduce unnecessary waiting time and subsequent trials.

**Study Design and Settings:**

A meta-epidemiologic study was conducted by collecting meta-analyses from the Cochrane Database of Systematic Reviews and five major medical journals. Effect size of a single trial was compared with that of its corresponding meta-analysis. The single trial includes the first trial, last trial and any trial randomly selected from the meta-analysis.

**Results:**

647 meta-analyses are included and the median number of trials in a meta-analysis is 5. 233 (36.0%) meta-analyses have the first trial with a statistically significant result. When the first trial is statistically significant, 84.1% (95% CI: 79.4%, 88.8%) of the corresponding meta-analyses is both in the same direction and statistically significant. When the first trial is statistically insignificant, 57.9% (95% CI: 53.2%, 62.8%) of the meta-analysis is also statistically insignificant regardless of direction. The median number of years is 6.5 years from the first to the 5^th^ trial.

**Conclusion:**

The conclusion of the first trial that the treatment is effective or harmful is mostly likely correct. A statistically significant trial agrees more often with its corresponding meta-analysis than a large trial. These findings imply that particularly in some urgent, life-saving or other critical circumstances for which no other effective methods are available, cautious recommendation based on the significant result of the first trial seems justifiable and could start use of an effective intervention by 5–8 years earlier.

## Introduction

Randomized controlled trials are generally viewed as the gold standard for evaluating the effectiveness of medical interventions [1]. The first trial on a topic, say the effectiveness of a new drug, is usually considered non-conclusive. Consequent trials are then conducted either to confirm the finding of the first trial in a similar condition or to see whether the finding may vary in different circumstances, such as in different ethnic groups of patients and/or in different care settings [2].

When a number of trials on a topic is accumulated and in particular when the trials are generally small, the meta-analysis is often used to combine the results of the individual trials in order to draw a more reliable conclusion supported by an increased statistical power [3]. As there are no clearly defined and widely agreed rules and methods to discontinue new trials even when the evidence is clearly sufficient, new studies may continue to be conducted as long as investigators like.

The major cost of the lack of stopping rules is time as clinical use of the tested intervention may have to wait for years or even over a decade before the so-called conclusive result from a meta-analysis of all trials become available [4], [5]. Nevertheless, in many cases, recommendations or guidelines are made based on a single large randomized clinical trial, experts' consensus, small studies, observational studies, or registries. For example, the latest guidelines on non-cardiac surgery stated that when no trials were available on a specific cardiac-management regimen in the surgical setting, data from the non-surgical setting were extrapolated, and similar recommendations were made, but with different levels of evidence [6].

The other major cost is resources spent on consequent trials that are unnecessary if the first trial (or the first few trials) has already provided reliable evidence for action.

The waste on conducting consequent trials and years of waiting could be large. For example, a cumulative meta-analysis in 1992 in the New England Journal of Medicine [7] showed that based on 20 trials and 6,935 subjects, intravenous streptokinase for acute myocardial infarction was shown clearly effective (P<0.001) before 1986. After that, 13 more randomized trials with a total of 30,039 patients were conducted, including two very large trials which had a total of 28,899 patients in them. However, the conclusion remained almost the same qualitatively and marginally different quantitatively. This seems to suggest 81% (30,039 out of 36,974) of the patients who were subjected to the trials are probably unnecessary. On the other hand, ineffective or even harmful treatments continued to be used in routine practice [7]–[10].

A recent study showed that the result from the first three trials would be good enough, implying clinical recommendations may not have to wait for so long for the meta-analysis [11]. Rapid reviews, that limit to particular aspects of and compromise in the breadth or depth of the systematic review process, have been proposed to provide “quick but not dirty” evidence [12]. These highlight the urgency for more speedy review and provision of evidence for clinical decision making.

In this study, we hypothesize that in some circumstances even the very first trial can well predict the result of meta-analysis and play an important role for practice in particular in some urgent, life-saving or other critical circumstances for which no effective treatments are available. Hence, the objective of this study is to explore how often and when the result of a single trial, in particular the first trial, would agree with that of its corresponding meta-analysis. As often a clinician may have only the most recent study or any convenient study in hand, we also examined how often these trials would predict the result of the meta-analysis.

## Materials and Methods

### Sources of meta-analyses

A meta-epidemiologic study was conducted and the meta-analyses of the current study were extracted from 2 sources, namely (i) 5 major medical journals including *New England Journal of Medicine (NEJM)*, *British Medical Journals (BMJ)*, *Annals of Internal Medicine (AIM)*, *Lancet*, and *Journal of American Medical Association (JAMA)*, and (ii) the *Cochrane Database of Systematic Reviews (CDSR)*. Systematic reviews in top medical journal tend to represent important or hot topics while those in the CDSR may provide a good complement and have more readily accessible data for analyses.

#### Major medical journals

PUBMED was searched on Jan 2013 to identify systematic reviews and meta-analyses in the five journals. Key words used in the search strategy included “systematic review”, “meta*analys*”, and “pooled analys*”, with restriction to the 5 journals and publications between 1 Jan 2001 and 31 Dec 2010. A total of 1,515 citations were identified and screened for eligibility based on their titles and abstracts. Full texts were downloaded for the included entries and scrutinized for identifying eligible systematic reviews.

#### Cochrane Database of Systematic Reviews

Information of all articles published in the CDSR before Jan 2013 was extracted from PUBMED using journal title search through Endnote. There were 8,600 published articles in the CDSR and 860 (10%) of them were randomly selected for the current study. The titles and abstracts of these 860 articles were first screened for eligibility. Then, full texts of the included articles were downloaded and screened for identifying relevant meta-analyses.

**Figure 1:**
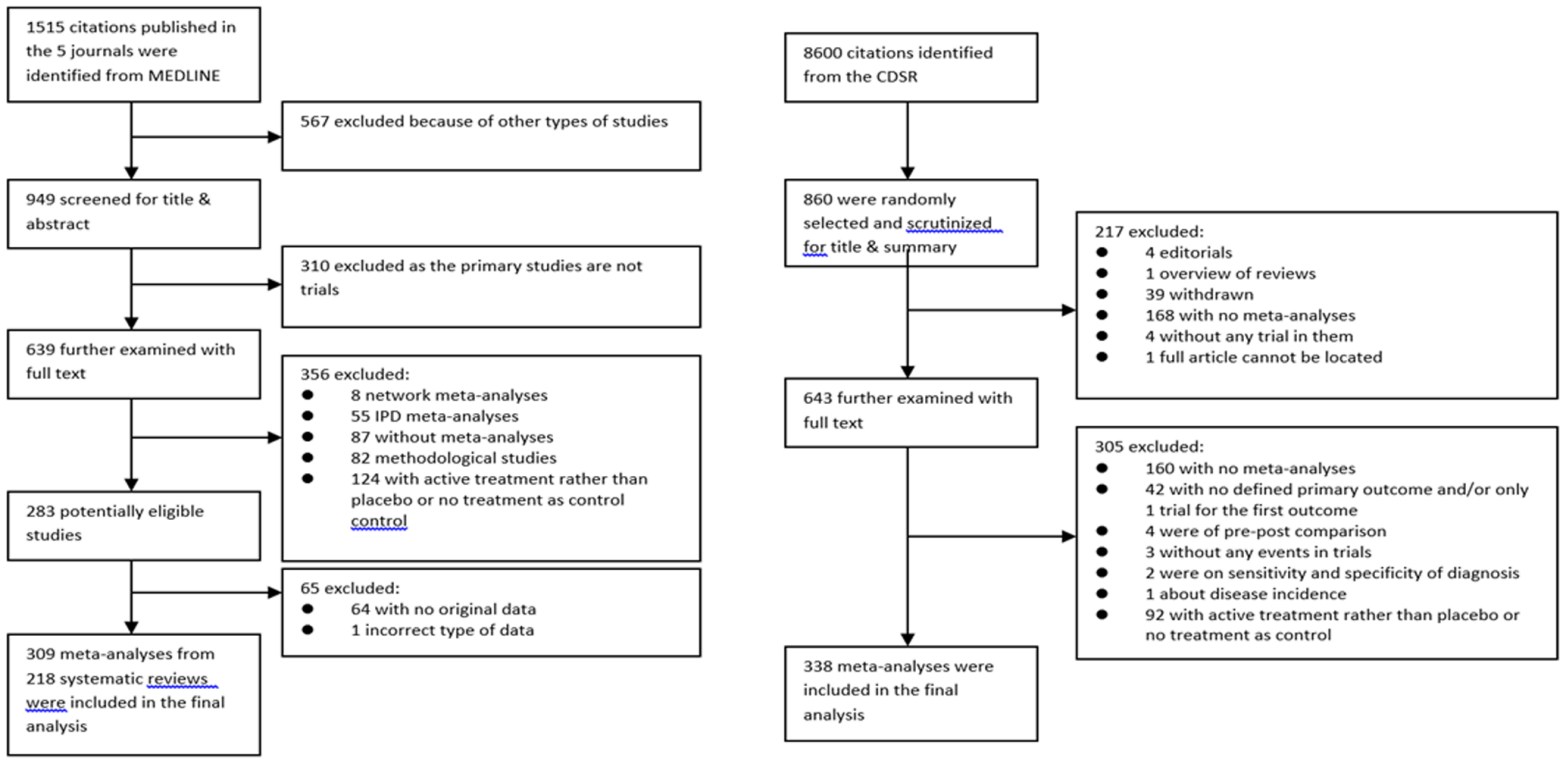
Flow chart of literature search.

Only systematic reviews of randomized controlled trials were included and the control group should be placebo, no intervention or usual care as the comparison treatment. Systematic reviews that compared two or more active treatments were excluded. In general, one meta-analysis was selected from one systematic review except the systematic review that compared two or more treatments with the same placebo or no treatment control, from which two or more meta-analyses may be included. The meta-analysis of primary outcome was included. If there were more than 1 primary outcome, hard outcome was selected. If the primary outcome was not clearly specified, the meta-analysis of the first outcome was used. The meta-analysis must have at least 2 trials in it. Only binary and continuous outcomes were considered for this study. The details of literature search and results at each step are shown in Figure 1.

### Data extraction

From each eligible meta-analysis included, the names of the first author and publication years of individual trials in the meta-analysis were extracted. For meta-analyses with a binary outcome, the number of subjects randomized and events in both the treatment and control group (i.e. the 2×2 table) were extracted for all trials. For meta-analyses with a continuous outcome, the total number of subjects, mean and standard error of both comparison groups were extracted. In case the raw data were not available, the effect and standard error of each trial were extracted.

### Statistical methods

The odds ratio was used as effect measure for all meta-analyses with a binary outcome and the mean difference or standardized mean difference for a continuous outcome depending on the effect measure used in the original meta-analysis. The trials in each meta-analysis were sorted according to the publication year in a chronological order and then the name of the first author in alphabetical order if there were 2 or more trials in a year. The first trial of a meta-analysis is defined as the earliest published study.

DerSimonian & Laird random effects model was used for combing the effects from individual studies [13]. Statistical significance was defined as p<0.05 unless otherwise specified differently.

The qualitative agreement between the first trial and its corresponding meta-analysis is defined as:

- When the effect of the first trial is statistically significant, it is considered in agreement with the meta-analysis qualitatively if the combined effect of the meta-analysis is also significant and in the same direction (i.e. both odds ratios are above or below one). Otherwise, the trial is considered to disagree with the meta-analysis qualitatively. This definition was used in comparing large trials and meta-analyses [14] but we combined in our analyses both “partially agree” and “disagree” into the “disagree” category. So our agreement is defined more stringently.
- When the effect of the first trial is statistically insignificant, it is considered in agreement with the meta-analysis qualitatively if the combined effect is also insignificant regardless its direction. Otherwise, the first trial is considered to disagree with the meta-analysis qualitatively. Although it is also important to know how often the single trial would agree with the meta-analysis when there is statistically insignificant and clearly lack of benefit, we did not define this category as it is subjective to define what constitutes lack of benefit.

We used statistical significance rather than confidence interval to define when the evidence is sufficient for action as the latter is context-dependent and further subject to personal judgment.

We stratified the meta-analyses according to the result of the significance test of the first trial into two categories: statistically significant (P≤0.05) and statistically insignificant (p>0.05). The agreement rate in the effect were computed and compared according to (i) the number of trials in the meta-analysis, (ii) the publication time of the first trial, (iii) the outcome type, and (iv) the effect size of the first trial in terms of the odds ratio. The mean difference and standardized mean difference were converted to an odds ratio by using the formula suggested by Borenstein [12]. The Chi-square test was used to examine the association between the agreement and it possible determining factors, while the Krustal-Wallis test was used to examine the difference of the medians between the categories of a factor. We also examined the ratio of the standard error of the meta-analysis to the first trial.

Sensitivity analyses were conducted by (i) changing the statistical significance for the first trial from p≤0.05 to p≤0.01, (ii) replacing the first trial by the last trial, and (iii) replacing the first trial by any randomly selected trial. For conducting (iii), we performed a simulation study in which we randomly selected a trial from each meta-analysis and compared its result with the meta-analysis. The whole process was repeated 10,000 times and hence 10,000 sets of results were obtained. The median agreement rates with their inter-quartile ranges were computed. The computation and simulation of meta-analyses were performed by using IML of Statistical Analysis System (SAS) [15] while the tests were conducted by using Proc FREQ and Proc NPAR1WAY in SAS.

## Results

Figure 1 shows the flow of literature search and publications identified at each step of the search. We scrutinized a total of 1515 citations from the 5 journals identified through MEDLINE and 860 citations randomly selected from all 8600 citations in the CDSR. 309 meta-analyses (extracted from 218 relevant systematic reviews) from the 5 journals and 338 from the CDSR were found eligible and included in the final analyses of this study. Details of the included systematic reviews are summarized in Table S1.

Table 1 summarized the characteristics of the 647 meta-analyses included in the study according to where they were identified. It is worth to note that the percentage of meta-analyses with the first trial statistically significant is similarly for both sources of systematic reviews: 36.6% for the journals and 35.5% for the CDSR. Meta-analyses from the journals tend to have a larger number of trials in each and their combined effects are more likely to be statistically significant than those from the CDSR. In total, 36.0% of meta-analyses have the first trial statistically significant and 57.2% of the combined effect of the meta-analyses is statistically significant. The median (Q1–Q3) number of trials in the meta-analyses with the significant and insignificant first trial are 5 (3–8) and 5 (3–9) respectively. From Mann-Whitney U-test, there is no significant difference between the number of trials in the two comparison groups (p = 0.870).

**Table 1:** Characteristics of the 647 meta-analyses according to where they are found.

Characteristics and difference between the 2 sources#	5 Journals (n = 309)	The CDSR (n = 338)	Total (n = 647)
Type of outcome (p = 0.596)			
• Binary	227 (73.5%)	242 (71.6%)	469 (72.5%)
• Continuous	82 (26.5%)	96 (28.4%)	178 (27.5%)
Number of trials in the meta-analysis (p<0.001)			
• 2–3	58 (18.8%)	148 (43.8%)	206 (31.8%)
• 4–7	119 (38.5%)	122 (36.1%)	241 (37.2%)
• 8 or more	132 (42.7%)	68 (20.1%)	200 (30.9%)
Publication year of the first trial (p = 0.017)			
• 1990 or before	126 (41.0%)	143 (42.3%)	269 (41.7%)
• 1991–2000	131 (42.7%)	114 (33.7%)	245 (38.0%)
• 2001 or later	50 (16.3%)	81 (24.0%)	131 (20.3%)
Number of years from the first to last trial (p = 0.408)			
• 0–5	86 (28.2%)	113 (33.5%)	199 (31.0%)
• 6–10	79 (25.9%)	89 (26.4%)	168 (26.2%)
• 11–15	54 (17.7%)	55 (16.3%)	109 (17.0%)
• 15 or more	86 (28.2%)	80 (23.7%)	166 (25.9%)
First trial significant (p = 0.778)			
• Yes	113 (36.6%)	120 (35.5%)	233 (36.0%)
• No	196 (63.4%)	218 (64.5%)	414 (64.0%)
Combined effect significant (p<0.001)			
• Yes	206 (66.7%)	164 (48.5%)	370 (57.2%)
• No	103 (33.3%)	174 (51.5%)	277 (42.8%)

# : p is the p-value for the Chi-square test for the difference of percentages between the 2 sources of meta-analyses.

Table 2 shows the agreement rate between the first trial and its corresponding meta-analysis. Overall, the first trial, if statistically significant, agrees with the meta-analysis in 196 or 84.1% of the cases (95% C.I.: 79.4%, 88.8%). In terms of the direction of the effect, the first trials agrees with 99.1% (97.9%, 100%) their corresponding meta-analyses.

**Table 2:** Agreement rate and relative difference in the odds ratio between the first trial and its corresponding meta-analysis.

Meta-analyses	First trial: significant			First trial: insignificant		
	No. of 1^st^ trials significant/meta-analyses	Median# absolute z-score of combined effect	Agreement Rate in % (95% C.I.)	No. of 1^st^ trials significant/meta-analyses	Median# absolute z-score of combined effect	Agreement Rate in % (95% C.I.)
Overall (647)	196/233	3.92	84.1 (79.4, 88.8)	240/414	1.52	57.9 (53.2, 62.8)
Number of trials						
*•2–3*	60/72	3.54	83.3 (74.7, 91.9)	104/134	0.94	77.6 (70.6, 84.7)
*•4–7*	77/90	3.83	85.6 (78.3, 92.8)	78/151	1.86	51.7 (43.7, 59.6)
*•8 or above*	59/71	4.36	83.1 (74.4, 91.8)	58/129	2.25	45.0 (36.4, 53.5)
		P_NP_ = 0.023	P_Chi_ = 0.892		P_NP_<0.001	P_Chi_<0.001*
Outcome type						
*•Binary*	118/144	3.88	81.9 (75.7, 88.2)	194/325	1.44	59.7 (54.3, 65.0)
*•Continuous*	78/89	3.97	87.6 (80.8, 94.4)	46/89	1.91	51.7 (41.3, 62.1)
		P_NP_ = 0.281	P_Chi_ = 0.248		P_NP_ = 0.071	P_Chi_ = 0.175
Publication year of the 1^st^ trial						
*•1990 or before*	76/96	4.04	79.2 (71.0, 87.2)	106/173	1.46	61.3 (54.0, 68.5)
*•1991–2000*	71/81	4.14	87.7 (80.5, 94.8)	86/164	1.87	52.4 (44.8, 60.0)
*•2001 or later*	49/56	3.69	87.5 (78.8, 96.2)	47/75	1.29	62.7 (51.7, 73.6)
		P_NP_ = 0.878	P_Chi_ = 0.892		P_NP_ = 0.046	P_Chi_ = 0.173
Effect size of the 1^st^ trial^Γ^						
*•1/3≤OR≤3*	59/69	3.86	85.5 (77.2, 93.8)	185/312	1.42	59.3 (53.8, 64.8)
*•OR<1/3 or OR>3*	121/147	3.70	82.3 (76.1, 88.5)	51/94	1.83	54.3 (44.2, 64.3)
		P_NP_ = 1.00	P_Chi_ = 0.557		P = 0.41	P_Chi_ = 0.385

P_Chi_ is the p-value from the Chi-square test, and* indicates the trend test is also significant; P_NP_ is the p-value of the non-parametric test for median;

# ^,^ median absolute z-score for the combined effect of the meta-analysis and the effect size of standardized mean difference or mean difference was converted to odds ratio by using standard formula.

When the first trial is statistically insignificant (n = 414), it agrees with the meta-analysis in 240 or 57.9% (95% C.I: 53.2%, 62.8%). For the remaining 174 meta-analyses with the first trial statistically insignificant, 155 (89.1%) were statistically significant and with the combined effect in the same direction as the first trial, and 19 (10.9%) were significant but with the combined effect in a direction different from the first trial.

For both significant and insignificant first trials, the agreement rate between the trial and meta-analysis remain very similar regardless of the number of trials in the meta-analysis, outcome variables used, year of publication of the first trial, and size of the effect of the first trial. One major exception is that when the first trial is insignificant, the agreement rate tends to decline considerably as the number of trials is increased.

In sensitivity analyses, we set the significance level for the first trial at p≤0.01 as the cutoff. The overall agreement rate is slightly increased from 84.1 to 85.4% (79.8%, 91.1%) if the first trial is significant and slightly decreased from 57.9% to 51.3% (46.9%, 55.7%) if the first trial is insignificant. Furthermore, if the first trial is replaced by the last trial or any randomly selected trial in the meta-analysis, the agreement remained largely the same. In particular, the overall agreement rate is 83.7% (inter-quartile range (IQR): 78.8%, 88.6%) if the last trial is significant and 56.8% (IQR: 52.1%, 61.5%) if the last trial is insignificant. The overall agreement rate is 83.0% (IQR: 81.9%, 84.1%) if the randomly selected trial is significant and 56.8% (IQR: 56.0%, 57.3%) if the selected trial is insignificant.

**Figure 2:**
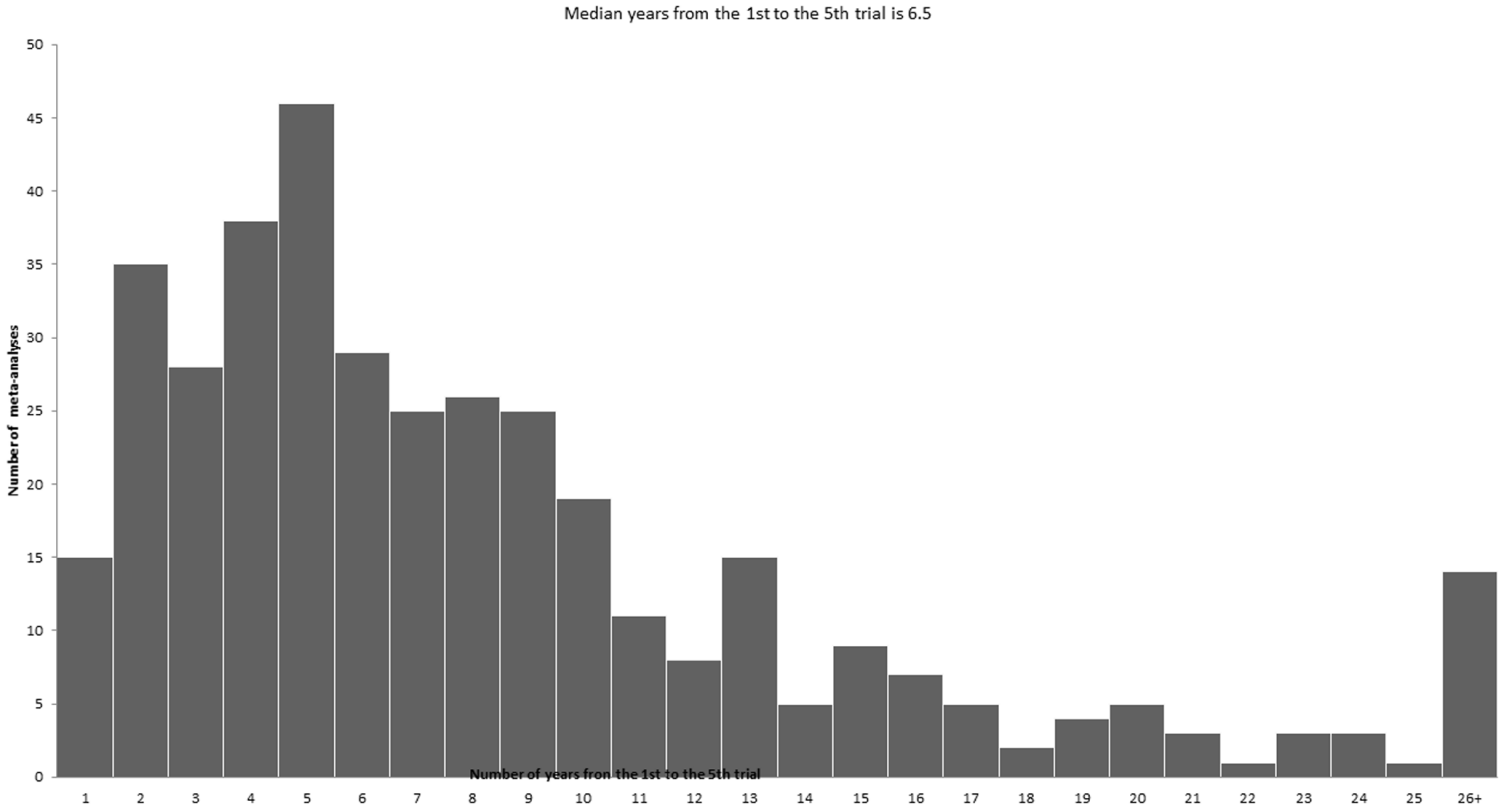
Number of years from the time of publication of the first trial to that of the fifth trial in the same meta-analysis. Only meta-analyses with 5 or more trials were eligible for this analysis.

Finally, Figure 2 showed the number of years that was needed from the first to the 5th in the meta-analyses with 5 trials or more in each. The median is 6.5 years from the first to the 5th trial. Similarly, we estimated that it took an average of 11 years from the first to the 10th trial (details not shown).

## Discussion

By reviewing 647 meta-analyses, we found that if the first trial (or the last trial or any trial in hand) reported a statistically significant result, its qualitative conclusion that the treatment is effective (or harmful) is very likely to be correct. These significant first trials represent some 36% of all the 647 meta-analyses examined in this study. When the first trial is significant, its effect is almost always in the same direction as that of the corresponding meta-analysis.

Importantly, the agreement between the first significant trial and its corresponding meta-analysis is as good as, if not better than, that between the large trial and its corresponding, which is 82% and 80% in studies conducted by Cappelleri [16] and LeLorier [17] respectively. Large trials generally are much more time-consuming and expensive to conduct than an average trial. On the other hand, if the first (or the last trial) reported a statistically insignificant result, the conclusions is equally likely to be wrong or right, implying in such a circumstance more trials would be needed in order to draw a valid conclusion.

For example, Spiedel and Bloom showed in 1983 as the first trial on the topic that psychosocial intervention was effective to reduce pain in patients with cancer [18]. Almost thirty years later after the first trial, Gorin and colleagues summarized in 2012 all 28 trials on the topic and concluded the intervention was effective. Given the unlikely adverse effect of such interventions, the treatment could have well been adopted more than twenty years ago based on the positive result of the first trial.

These results raise an important question about the current unsystematic approach to getting research evidence to practice. Meta-analysis is widely believed to provide the best evidence for medical decisions and often required by clinicians and guidelines and policy makers [19]. However, when the first trial is statistically significant, new trials are unlikely to change the conclusion of the first trial whether the treatment be effective (most of time) or harmful (occasionally).

Having said that, it is likely that an intervention may be more effective in some circumstances than others and subsequent trials are needed to address this issue. However, it is very likely that such clinical heterogeneity is suspected only many years after the treatment has been used in practice. Put it differently, the argument for testing clinical heterogeneity should not be used as an excuse for no action when the first trial is statistically significant. The contrary is probably true: the earlier an effective treatment is introduced into practice, the sooner clinical heterogeneity can be detected and subjected to further evaluation in trials.

Moreover, our study also showed it could take a median of 6.5 years before 5 trials in a topic can cumulate and 11 years before 10 trials can accumulate. In extreme cases, new trials are still being conducted some 30 years after the first trial showed clearly the treatment was effective [7], [8]. There is generally no consensus on how many trials would be required in a meta-analysis before a reliable conclusion can be drawn in a meta-analysis. Given the fact that clinical decisions are often made based only on the qualitative information in which treatments are divided into effective and ineffective, it seems recommendation based on the first trial with a statistically significant result is justified and will be mostly correct. Nevertheless, it is clear that each recommendation should be considered independently and sometimes quantitative information on the effectiveness is critical in decision making.

In addition, our study also showed that the standard error of the meta-analysis would be around half (ie, 53%) of that of the first significant trial. In other words, the width of the 95% confidence interval of the combined estimate would be around half of the width of the 95% C.I. from the first trial.

Furthermore, often what the physician has got in hand is the most recently published trial (which we call the last trial). Again, if the last trial conveniently available showed a statistically significant result, the conclusion that the treatment is effective or harmful is very likely to be as good as that from the meta-analysis that combines all the relevant studies. In such circumstances, the clinician should feel almost as confident as having a meta-analysis in hand, which will save the effort of finding the meta-analysis or more trials.

Our study also showed that if the first trial showed a statistically significant result, a meta-analysis of the first few trials would be sufficient to draw a conclusion that is as good as that of a meta-analysis of 8 trials or more. This is largely consistent with the results of a recent study that showed first five trials combined would be good enough to draw a valid qualitative conclusion [11]. This showed that, in many cases, only a few trials were necessary before getting a reasonably robust answer and, in most cases, five or more trials would be all that is needed to give good confidence that the answer is valid, but it is difficult to predict when the result of the meta-analysis will change and it will not. Our result suggested that, if the first trial is significant, the result of the meta-analysis is very likely be significant and in the same direction. More conveniently for decision makers, any significant trial in had would be able to predict very reliably the result of future meta-analysis.

Some would argue that the first significant trial predicts well the result of the corresponding meta-analysis may be partly because positive trials tend to attract more attention and subsequent studies to confirm the results. However, we found the median, lower quartile and upper quartile of the number of trials included in the meta-analyses for the first positive and negative trials are not significantly difference.

These findings seem to land further support for the idea of rapid reviews in which a quick search of literature that can identify a few relevant trials without exhausting all relevant ones would be good enough. More studies are deserved to further look into the issue.

One the other hand, when the first trial is insignificant, 57.9% of the corresponding meta-analyses would also be insignificant or, put it differently, 42.1% of the corresponding meta-analysis would become statistically significant. It emphasizes the message that when the first trial is statistically not significant, there is still over 40% chance that the combined result from many trials can be significant. This is exactly the reason for conducting meta-analyses and increasing the power by combining many small individual studies. For example, Furukawa and colleagues [20] conducted a systematic review and meta-analysis involving 1160 subjects on the effect of low dosage tricyclic antidepressants in depression and concluded the effect was statistically significantly better than placebo. The first study in the meta-analysis was conducted by Tetreault and colleagues in 1966 [21] involving only 22 subjects but the result was however statistically non-significant. Intuitively, our study suggests that the type I error of using the first trial to predict the corresponding meta-analysis is 16%

This analysis is based on 647 meta-analyses. We included any meta-analyses with 2 or more trials. The meta-analyses are from different fields and hence represent various clinical situations. The odds ratio was used instead of risk difference for binary outcome since relative effect measures tend to be more homogeneous [22].

There are some concerns about the validity of the findings. First, the first trials from meta-analyses used in this study do not include those for which no meta-analyses have been conducted. It is probably reasonable to hypothesize that the first trials that have a very large and conclusive effect would in general discourage more trials on the topic to follow. Furthermore, if indeed a few trials have been conducted and shown conclusively a large effect, a meta-analysis may not be conducted. As a result, the first trials we included may have relatively a smaller and less conclusive effect than all the first trials. If this is true, the agreement rate between the first significant trials and its corresponding meta-analyses would be probably even higher than we observed in this study.

Second, it is also possible that the effect of the first trial may be in a different direction from the meta-analysis, namely the first significant trial showed the treatment was effective but the meta-analysis showed it is harmful, or vice versa. Such cases however did not exist in the 647 meta-analyses we examined.

Third, due to selective publication it is likely that subsequent trials with negative results are less likely to be published than positive trials when the first trial is positive [23], [24], which may have made the meta-analysis more likely to agree with the first significant trial. However, we have not found any empirical evidence for a fair judgment about the possible bias thus induced. However, significant results of meta-analyses might be more welcome by journal editors and hence publication bias of meta-analyses cannot be ruled out.

Moreover, when no primary outcome was specified in the systematic review, we used the first reported meta-analysis in the review. It is possible that the first reported meta-analysis is likely to be statistically significant and hence selection bias may result, although we have not seen direct evidence to support this assumption. Defining primary outcome(s) for systematic reviews and meta-analyses is required by the Cochrane Collaboration [25] and suggested by the QUOROM Statement [26] as well as PRISMA Guidelines [27]. Thus, it is true that an increasing number ofsystematic reviews published after 2000 defined their primary outcomes.

Before we conclude, one important issue must be re-visited. That is when the evidence on the effectiveness of a treatment would be sufficiently good so that recommendation is justified. A significant trial has 80–90% chance of agreeing with the meta-analysis. Is this good enough? Are additional trials and meta-analysis still necessary in such a case? It probably depends. The best is the enemy of the good. If we wait for the best, we may never be able to act as definite evidence can rarely or never be reached as regards the effectiveness of medical interventions. Even a higher certainty such as 95% will be still an arbitrary choice.

85% certainty is in many medical circumstances probably good enough for action. For example, Spiegel and Bloom showed in 1983 as the first trial of its kind that psychosocial intervention was effective to reduce pain in patients with cancer [18]. Twenty-years later since the first trial, Gorin and colleagues summarized in 2012 all 28 trials on this topic and concluded the intervention was effective. Given the unlikely of adverse effect of such interventions, the treatment could have well been adopted 29 years ago. However, it needs to be born in mind that there is still 15% chance that the treatment can be ineffective and hence cautions should be taken wherever deemed necessary.

Real practice seems also to be in line with our conclusion. For example, the US FDA normally does not require for a meta-analysis for approving a new drug: the first high quality, positive trial(s) are all needed. At most, such trials have a certainty of some 85% as we demonstrated. Furthermore, by using less stringent agreement criteria than ours, JAMA in 1996 [16] and NEJM in 1997 [17] found only some 80% large trials agreed with meta-analyses; large trials which are often highly emphasized agree with meta-analyses less often than single significant trials. Clinical Evidence showed that in real practice, some 50% of commonly used interventions are of uncertain effect and continue to be widely used.

In conclusion, the qualitative conclusion of the first trial or any significant trial conveniently in hand that a treatment is effective or harmful is most likely correct and the true effectiveness is probably even larger. In this case, recommendations based on the first significant trial seems justified particularly in some urgent, life-saving or other critical circumstances, such as SARS and Ebola virus infection, for which currently no effective treatments are available and it could start introduction of an effective intervention many years earlier. Conversely, when a trial is statistically insignificant, more trials would be needed in order to draw a valid conclusion.

Our findings also call for attention to a more general question when research evidence should be considered sufficient for action and support the need for further exploration of the idea of rapid reviews which may speed up the review and provision of evidence for medical decision making.

## Supporting Information

Table S1 Reference list of included systematic reviews.
(DOCX)

## References

[1] Kaptchuk TJ (2001) The double-blind, randomized, placebo-controlled trial: gold standard or golden calf? J Clin Epidemiol 54:541–549. 11377113 10.1016/s0895-4356(00)00347-4

[2] Baum M, Houghton J (1999) Contribution of randomised controlled trials to understanding and management of early breast cancer. BMJ 319:568–571. 10463904 10.1136/bmj.319.7209.568 PMC1116442

[3] Straus SE (2005) Evidence-based medicine: how to practice and teach EBM. Edinburgh; New York: Elsevier/Churchill Livingstone. p. p.

[4] Hailey D, Corabian P, Harstall C, Schneider W (2000) The use and impact of rapid health technology assessments. Int J Technol Assess Health Care 16:651–656. 10932429 10.1017/s0266462300101205

[5] Thompson SG, Sharp SJ (1999) Explaining heterogeneity in meta-analysis: a comparison of methods. Stat Med 18:2693–2708. 10521860 10.1002/(sici)1097-0258(19991030)18:20<2693::aid-sim235>3.0.co;2-v

[6] Kristensen SD, Knuuti J, Saraste A, Anker S, Botker HE, et al (2014) 2014 ESC/ESA Guidelines on non-cardiac surgery: cardiovascular assessment and management: The Joint Task Force on non-cardiac surgery: cardiovascular assessment and management of the European Society of Cardiology (ESC) and the European Society of Anaesthesiology (ESA). Eur J Anaesthesiol 10.1097/EJA.0000000000000150. 10.1097/EJA.0000000000000150 25127426

[7] Lau J, Antman EM, Jimenez-Silva J, Kupelnick B, Mosteller F, et al (1992) Cumulative meta-analysis of therapeutic trials for myocardial infarction. N Engl J Med 327:248–254. 1614465 10.1056/NEJM199207233270406

[8] Antman EM, Lau J, Kupelnick B, Mosteller F, Chalmers TC (1992) A comparison of results of meta-analyses of randomized control trials and recommendations of clinical experts. Treatments for myocardial infarction. JAMA 268:240–248. 1535110

[9] Luo XM, Tang JL, Hu YH, Li LM, Wang YL, et al (2013) How often are ineffective interventions still used in clinical practice? A cross-sectional survey of 6,272 clinicians in China. PLoS One 8:e52159. 23533565 10.1371/journal.pone.0052159 PMC3606390

[10] Rennie D, Chalmers I (2009) Assessing authority. JAMA 301:1819–1821. 19417202 10.1001/jama.2009.559

[11] Herbison P, Hay-Smith J, Gillespie WJ (2011) Meta-analyses of small numbers of trials often agree with longer-term results. J Clin Epidemiol 64:145–153. 20609563 10.1016/j.jclinepi.2010.02.017

[12] Grant MJ, Booth A (2009) A typology of reviews: an analysis of 14 review types and associated methodologies. Health Info Libr J 26:91–108. 19490148 10.1111/j.1471-1842.2009.00848.x

[13] Borenstein M (2009) Introduction to meta-analysis. Chichester, U.K.: John Wiley & Sons. xxviii, 421 p. p.

[14] Villar J, Carroli G, Belizan JM (1995) Predictive ability of meta-analyses of randomised controlled trials. Lancet 345:772–776. 7891492 10.1016/s0140-6736(95)90646-0

[15] SAS Institute (2004) SAS/IML 9.1: user's guide. Cary, NC: SAS Pub.

[16] Cappelleri JC, Ioannidis JP, Schmid CH, de Ferranti SD, Aubert M, et al (1996) Large trials vs meta-analysis of smaller trials: how do their results compare? JAMA 276:1332–1338. 8861993

[17] LeLorier J, Gregoire G, Benhaddad A, Lapierre J, Derderian F (1997) Discrepancies between meta-analyses and subsequent large randomized, controlled trials. N Engl J Med 337:536–542. 9262498 10.1056/NEJM199708213370806

[18] Spiegel D, Bloom JR (1983) Group therapy and hypnosis reduce metastatic breast carcinoma pain. Psychosom Med 45:333–339. 6622622 10.1097/00006842-198308000-00007

[19] Cook DJ, Mulrow CD, Haynes RB (1997) Systematic reviews: synthesis of best evidence for clinical decisions. Ann Intern Med 126:376–380. 9054282 10.7326/0003-4819-126-5-199703010-00006

[20] Furukawa TA, McGuire H, Barbui C (2002) Meta-analysis of effects and side effects of low dosage tricyclic antidepressants in depression: systematic review. BMJ 325:991. 12411354 10.1136/bmj.325.7371.991 PMC131022

[21] Tetreault L, Doucet P, Blanchet A, Bordeleau JM (1966) [Comparative evaluation of the antidepressive properties of opipramol, imipramine and placebo in neurotic depression]. Union Med Can 95:546–553. 5326793

[22] Walter SD (2000) Choice of effect measure for epidemiological data. J Clin Epidemiol 53:931–939. 11004419 10.1016/s0895-4356(00)00210-9

[23] Hopewell S, Clarke M, Stewart L, Tierney J (2007) Time to publication for results of clinical trials. Cochrane Database Syst Rev 10.1002/14651858.MR000011.pub2: MR000011. 10.1002/14651858.MR000011.pub2 PMC7437393 17443632

[24] Sutton AJ, Duval SJ, Tweedie RL, Abrams KR, Jones DR (2000) Empirical assessment of effect of publication bias on meta-analyses. BMJ 320:1574–1577. 10845965 10.1136/bmj.320.7249.1574 PMC27401

[25] Higgins JPT, Green SEditors (2005) Cochrane Handbook for Systematic Reviews of Interventions 4.2.4. Chichester, UK: John Wiley & Sons, Ltd. 239 p.

[26] Moher D, Cook DJ, Eastwood S, Olkin I, Rennie D, et al (1999) Improving the quality of reports of meta-analyses of randomised controlled trials: the QUOROM statement. Quality of Reporting of Meta-analyses. Lancet 354:1896–1900. 10584742 10.1016/s0140-6736(99)04149-5

[27] Moher D, Liberati A, Tetzlaff J, Altman DG, Group P (2009) Preferred reporting items for systematic reviews and meta-analyses: the PRISMA statement. J Clin Epidemiol 62:1006–1012. 19631508 10.1016/j.jclinepi.2009.06.005

