# Supplementary material for: How Often Does an Individual Trial Agree with Its Corresponding Meta-Analysis? A Meta-Epidemiologic Study
Source: PLoS One. 2014 Dec 4;9(12):e113994. doi: 10.1371/journal.pone.0113994 (PMC4256383; doi:10.1371/journal.pone.0113994)
Supplement: Table S1 — Reference list of included systematic reviews. (DOCX) [file pone.0113994.s001.docx]

Table S1: List of included systematic reviews

From major medical journals:

Aasbo JD, Lawrence AT, Krishnan K, Kim MH, Trohman RG. (2005) Amiodarone prophylaxis reduces major cardiovascular morbidity and length of stay after cardiac surgery: a meta-analysis. Ann Intern Med 143:327-36.

Adhikari NK, Burns KE, Friedrich JO, Granton JT, Cook DJ, et al. (2007) Effect of nitric oxide on oxygenation and mortality in acute lung injury: systematic review and meta-analysis. BMJ 334:779. Epub 2007 Mar 23.

Adu D, Cockwell P, Ives NJ, Shaw J, Wheatley K. (2003) Interleukin-2 receptor monoclonal antibodies in renal transplantation: meta-analysis of randomised trials. BMJ 326:789.

Advanced Bladder Cancer Meta-analysis Collaboration (2003) .Neoadjuvant chemotherapy in invasive bladder cancer: a systematic review and meta-analysis. Lancet 361:1927-34.

Afshari A, Wetterslev J, Brok J, Møller A. (2007) Antithrombin III in critically ill patients: systematic review with meta-analysis and trial sequential analysis. BMJ 335:1248-51. Epub 2007 Nov 23.

Als-Nielsen B, Gluud LL, Gluud C. (2004) Non-absorbable disaccharides for hepatic encephalopathy: systematic review of randomised trials. BMJ 328: 1046. Epub 2004 Mar 30.

Amori RE, Lau J, (2007) Pittas AG.Efficacy and safety of incretin therapy in type 2 diabetes: systematic review and meta-analysis. JAMA 298: 194-206.

Andraws R, Berger JS, Brown DL. (2005) Effects of antibiotic therapy on outcomes of patients with coronary artery disease: a meta-analysis of randomized controlled trials. JAMA 293: 2641-7.

Annane D, Bellissant E, Bollaert PE, Briegel J, Confalonieri M, et al. (2009) Corticosteroids in the treatment of severe sepsis and septic shock in adults: a systematic review. JAMA 301: 2362-75. doi: 10.1001/jama.2009.815.

Annane D, Bellissant E, Bollaert PE, Briegel J, Keh D, et al. (2004) Corticosteroids for severe sepsis and septic shock: a systematic review and meta-analysis. BMJ 329: 480. Epub 2004 Aug 2.

Aponte JJ, Schellenberg D, Egan A, Breckenridge A, Carneiro I, et al. (2009) Efficacy and safety of intermittent preventive treatment with sulfadoxine-pyrimethamine for malaria in African infants: a pooled analysis of six randomised, placebo-controlled trials. Lancet 374: 1533-42. doi: 10.1016/S0140-6736(09)61258-7. Epub 2009 Sep 16.

Arroll B, Goodyear-Smith F. (2004) Corticosteroid injections for osteoarthritis of the knee: meta-analysis. BMJ 328: 869. Epub 2004 Mar 23.

Arroll B, Kenealy T. (2006) Are antibiotics effective for acute purulent rhinitis? Systematic review and meta-analysis of placebo controlled randomised trials. BMJ 333: 279. Epub 2006 Jul 21.

Ashcroft DM, Dimmock P, Garside R, Stein K, Williams HC. (2005) Efficacy and tolerability of topical pimecrolimus and tacrolimus in the treatment of atopic dermatitis: meta-analysis of randomised controlled trials. BMJ 330: 516. Epub 2005 Feb 24

ASTEC/EN.5 Study Group, Blake P, Swart AM, Orton J, Kitchener H, et al. (2009) Adjuvant external beam radiotherapy in the treatment of endometrial cancer (MRC ASTEC and NCIC CTG EN.5 randomised trials): pooled trial results, systematic review, and meta-analysis. Lancet 373: 137-46. doi: 10.1016/S0140-6736(08)61767-5. Epub 2008 Dec 16.

Avni T, Levcovich A, Ad-El DD, Leibovici L, Paul M. (2010) Prophylactic antibiotics for burns patients: systematic review and meta-analysis. BMJ 340: c241. doi: 10.1136/bmj.c241.

Bachmann S, Finger C, Huss A, Egger M, Stuck AE, et al. (2010) Inpatient rehabilitation specifically designed for geriatric patients: systematic review and meta-analysis of randomised controlled trials. BMJ 340:c1718. doi: 10.1136/bmj.c1718.

Baker WL, Coleman CI, Kluger J, Reinhart KM, Talati R, et al. (2009) Systematic review: comparative effectiveness of angiotensin-converting enzyme inhibitors or angiotensin II-receptor blockers for ischemic heart disease.Ann Intern Med. 151(12):861-71. doi: 10.7326/0003-4819-151-12-200912150-00162.

Bangalore S, Wetterslev J, Pranesh S, Sawhney S, Gluud C, et al. (2008) Perioperative beta blockers in patients having non-cardiac surgery: a meta-analysis.Lancet. 372:1962-76. doi: 10.1016/S0140-6736(08)61560-3. Epub 2008 Nov 13.

Barr RG, Rowe BH, Camargo CA Jr. (2003) Methylxanthines for exacerbations of chronic obstructive pulmonary disease: meta-analysis of randomised trials. BMJ 327(7416):643. Erratum in: BMJ. 2003 Oct 18;327(7420):919.

Bateman E, Nelson H, Bousquet J, Kral K, Sutton L, et al. (2008) Meta-analysis: effects of adding salmeterol to inhaled corticosteroids on serious asthma-related events. Ann Intern Med. 149: 33-42. Epub 2008 Jun 3.

Bazzano LA, Reynolds K, Holder KN, He J. (2006) Effect of folic acid supplementation on risk of cardiovascular diseases: a meta-analysis of randomized controlled trials. JAMA 2006 Dec 13; 296:2720-6.

Bennett CL, Silver SM, Djulbegovic B, Samaras AT, Blau CA, et al. (2008) Venous thromboembolism and mortality associated with recombinant erythropoietin and darbepoetin administration for the treatment of cancer-associated anemia. JAMA 299: 914-24. doi: 10.1001/jama.299.8.914.

Berger JS, Roncaglioni MC, Avanzini F, Pangrazzi I, Tognoni G, et al. (2006) Aspirin for the primary prevention of cardiovascular events in women and men: a sex-specific meta-analysis of randomized controlled trials. JAMA 295: 306-13.

Beswick AD, Rees K, Dieppe P, Ayis S, Gooberman-Hill R, et al. (2008) Complex interventions to improve physical function and maintain independent living in elderly people: a systematic review and meta-analysis. Lancet 371: 725-35. doi: 10.1016/S0140-6736(08)60342-6.

Birck R, Krzossok S, Markowetz F, Schnülle P, van der Woude FJ, et al. (2003) Acetylcysteine for prevention of contrast nephropathy: meta-analysis. Lancet 362: 598-603.

Bischoff-Ferrari HA, Dawson-Hughes B, Staehelin HB, Orav JE, Stuck AE, et al. (2009) Fall prevention with supplemental and active forms of vitamin D: a meta-analysis of randomised controlled trials. BMJ 339:b3692. doi: 10.1136/bmj.b3692.

Bischoff-Ferrari HA, Dawson-Hughes B, Willett WC, Staehelin HB, Bazemore MG, et al. (2004) Effect of Vitamin D on falls: a meta-analysis. JAMA 291: 1999-2006.

Bischoff-Ferrari HA, Willett WC, Wong JB, Giovannucci E, Dietrich T, et al. (2005) Fracture prevention with vitamin D supplementation: a meta-analysis of randomized controlled trials. JAMA 293: 2257-64.

Bjelakovic G, Nikolova D, Gluud LL, Simonetti RG, Gluud C. (2007) Mortality in randomized trials of antioxidant supplements for primary and secondary prevention: systematic review and meta-analysis. JAMA 297: 842-57.

Bjelakovic G, Nikolova D, Simonetti RG, Gluud C. (2004) Antioxidant supplements for prevention of gastrointestinal cancers: a systematic review and meta-analysis. Lancet 364:1219-28.

Bjordal JM, Ljunggren AE, Klovning A, Slørdal L. (2004) Non-steroidal anti-inflammatory drugs, including cyclo-oxygenase-2 inhibitors, in osteoarthritic knee pain: meta-analysis of randomised placebo controlled trials. BMJ 329: 1317. Epub 2004 Nov 23.

Bongartz T, Sutton AJ, Sweeting MJ, Buchan I, Matteson EL, et al. (2006) Anti-TNF antibody therapy in rheumatoid arthritis and the risk of serious infections and malignancies: systematic review and meta-analysis of rare harmful effects in randomized controlled trials. JAMA 295: 2275-85.

Boulé NG, Haddad E, Kenny GP, Wells GA, Sigal RJ. (2001) Effects of exercise on glycemic control and body mass in type 2 diabetes mellitus: a meta-analysis of controlled clinical trials. JAMA 286:1218-27.

Bradley DJ, Bradley EA, Baughman KL, Berger RD, Calkins H, et al. (2003) Cardiac resynchronization and death from progressive heart failure: a meta-analysis of randomized controlled trials. JAMA 289:730-40.

Brewster LM, van Montfrans GA, Kleijnen J. (2004) Systematic review: antihypertensive drug therapy in black patients. Ann Intern Med 141:614-27.

Bridge JA, Iyengar S, Salary CB, Barbe RP, Birmaher B, et al. (2007) Clinical response and risk for reported suicidal ideation and suicide attempts in pediatric antidepressant treatment: a meta-analysis of randomized controlled trials. JAMA 297:1683-96.

Briel M, Schwartz GG, Thompson PL, de Lemos JA, Blazing MA, et al. (2006) Effects of early treatment with statins on short-term clinical outcomes in acute coronary syndromes: a meta-analysis of randomized controlled trials. JAMA 295: 2046-56.

Briel M, Schwartz GG, Thompson PL, de Lemos JA, Blazing MA, et al. (2006) Effects of early treatment with statins on short-term clinical outcomes in acute coronary syndromes: a meta-analysis of randomized controlled trials. JAMA 295: 2046-56.

Brophy JM, Joseph L, Rouleau JL. (2001) Beta-blockers in congestive heart failure. A Bayesian meta-analysis. Ann Intern Med 134:550-60.

Buscemi N, Vandermeer B, Hooton N, Pandya R, Tjosvold L, et al. (2006) Efficacy and safety of exogenous melatonin for secondary sleep disorders and sleep disorders accompanying sleep restriction: meta-analysis. BMJ 332: 385-93. Epub 2006 Feb 10.

Busse JW, Kaur J, Mollon B, Bhandari M, Tornetta P 3rd, et al. (2009) Low intensity pulsed ultrasonography for fractures: systematic review of randomised controlled trials. BMJ 338:b351. doi: 10.1136/bmj.b351

Carlberg B, Samuelsson O, Lindholm LH. (2005) Atenolol in hypertension: is it a wise choice? Lancet 364: 1684-9. Erratum in: Lancet 365: 656.

Caughey AB, Sundaram V, Kaimal AJ, Gienger A, Cheng YW, et al. (2009) Systematic review: elective induction of labor versus expectant management of pregnancy. Ann Intern Med 151: 252-63, W53-63.

Chan EY, Ruest A, Meade MO, Cook DJ. (2007) Oral decontamination for prevention of pneumonia in mechanically ventilated adults: systematic review and meta-analysis. BMJ 334: 889. Epub 2007 Mar 26.

Chang AB, Lasserson TJ, Kiljander TO, Connor FL, Gaffney JT, et al. (2006) Systematic review and meta-analysis of randomised controlled trials of gastro-oesophageal reflux interventions for chronic cough associated with gastro-oesophageal reflux. BMJ 332: 11-7. Epub 2005 Dec 5.

Chou R, Fu R, Carrino JA, Deyo RA. (2009) Imaging strategies for low-back pain: systematic review and meta-analysis. Lancet 373:463-72. doi: 10.1016/S0140-6736(09)60172-0.

Chow RT, Johnson MI, Lopes-Martins RA, Bjordal JM. (2009) Efficacy of low-level laser therapy in the management of neck pain: a systematic review and meta-analysis of randomised placebo or active-treatment controlled trials. Lancet 374: 1897-908. doi: 10.1016/S0140-6736(09)61522-1. Epub 2009 Nov 13.

Christensen R, Kristensen PK, Bartels EM, Bliddal H, Astrup A. (2007) Efficacy and safety of the weight-loss drug rimonabant: a meta-analysis of randomised trials. Lancet 370: 1706-13. Review. Erratum in: Lancet 371: 558.

Chung M, Raman G, Trikalinos T, Lau J, Ip S. (2008) Interventions in primary care to promote breastfeeding: an evidence review for the U.S. Preventive Services Task Force. Ann Intern Med 149 :565-82.

Clark AM, Hartling L, Vandermeer B, McAlister FA. (2005) Meta-analysis: secondary prevention programs for patients with coronary artery disease. Ann Intern Med 143: 659-72.

Clark CE, Smith LF, Taylor RS, Campbell JL. (2010) Nurse led interventions to improve control of blood pressure in people with hypertension: systematic review and meta-analysis. BMJ 341: c3995. doi: 10.1136/bmj.c3995.

Clark RA, Inglis SC, McAlister FA, Cleland JG, Stewart S. (2007) Telemonitoring or structured telephone support programmes for patients with chronic heart failure: systematic review and meta-analysis. BMJ 334: 942. Epub 2007 Apr 10.

Coker TR, Chan LS, Newberry SJ, Limbos MA, Suttorp MJ, et al. (2010) Diagnosis, microbial epidemiology, and antibiotic treatment of acute otitis media in children: a systematic review. JAMA 304:2161-9. doi: 10.1001/jama.2010.1651.

Colfax G, Santos GM, Chu P, Vittinghoff E, Pluddemann A, et al. (2010) Amphetamine-group substances and HIV. Lancet 376: 458-74. doi: 10.1016/S0140-6736(10)60753-2.

Colman I, Friedman BW, Brown MD, Innes GD, Grafstein E, et al. (2008) Parenteral dexamethasone for acute severe migraine headache: meta-analysis of randomised controlled trials for preventing recurrence. BMJ 336: 1359-61. doi: 10.1136/bmj.39566.806725.BE. Epub 2008 Jun 9

Colman I, Brown MD, Innes GD, Grafstein E, Roberts TE, et al. (2004) Parenteral metoclopramide for acute migraine: meta-analysis of randomised controlled trials. BMJ 329: 1369-73. Epub 2004 Nov 18.

Colorectal Cancer Collaborative Group. (2001) Adjuvant radiotherapy for rectal cancer: a systematic overview of 8,507 patients from 22 randomised trials. Lancet 358: 1291-304.

Coombes BK1, Bisset L, Vicenzino B. (2010) Efficacy and safety of corticosteroid injections and other injections for management of tendinopathy: a systematic review of randomised controlled trials. Lancet 376: 1751-67. doi: 10.1016/S0140-6736(10)61160-9. Epub 2010 Oct 21.

Cooper NJ, Sutton AJ, Abrams KR, Wailoo A, Turner D, et al. (2003) Effectiveness of neuraminidase inhibitors in treatment and prevention of influenza A and B: systematic review and meta-analyses of randomised controlled trials. BMJ 326: 1235.

Costa J, Borges M, David C, Vaz Carneiro A. (2006) Efficacy of lipid lowering drug treatment for diabetic and non-diabetic patients: meta-analysis of randomised controlled trials. BMJ 332:1115-24. Epub 2006 Apr 3.

Cummings KJ, Lee SM, West ES, Cid-Ruzafa J, Fein SG, et al. (2001) Interferon and ribavirin vs interferon alone in the re-treatment of chronic hepatitis C previously nonresponsive to interferon: A meta-analysis of randomized trials. JAMA 285: 193-9.

Dagenais GR, Pogue J, Fox K, Simoons ML, Yusuf S. (2006) Angiotensin-converting-enzyme inhibitors in stable vascular disease without left ventricular systolic dysfunction or heart failure: a combined analysis of three trials. Lancet 368: 581-8.

de Almeida JR, Al Khabori M, Guyatt GH, Witterick IJ, Lin VY, et al. (2009) Combined corticosteroid and antiviral treatment for Bell palsy: a systematic review and meta-analysis. JAMA 302: 985-93. doi: 10.1001/jama.2009.1243.

Deeks JJ, Smith LA, Bradley MD. (2002) Efficacy, tolerability, and upper gastrointestinal safety of celecoxib for treatment of osteoarthritis and rheumatoid arthritis: systematic review of randomised controlled trials. BMJ 325: 619.

Del Brutto OH, Roos KL, Coffey CS, García HH. (2006) Meta-analysis: Cysticidal drugs for neurocysticercosis: albendazole and praziquantel. Ann Intern Med 145: 43-51.

Delbaldo C, Michiels S, Syz N, Soria JC, Le Chevalier T, et al. (2004) Benefits of adding a drug to a single-agent or a 2-agent chemotherapy regimen in advanced non-small-cell lung cancer: a meta-analysis. JAMA 292: 470-84.

Dennis CL. (2005) Psychosocial and psychological interventions for prevention of postnatal depression: systematic review. BMJ 331: 15.

Dentali F, Douketis JD, Gianni M, Lim W, Crowther MA. (2007) Meta-analysis: anticoagulant prophylaxis to prevent symptomatic venous thromboembolism in hospitalized medical patients. Ann Intern Med 146: 278-88.

Deshpande G, Rao S, Patole S. (2007) Probiotics for prevention of necrotising enterocolitis in preterm neonates with very low birthweight: a systematic review of randomised controlled trials. Lancet 369: 1614-20.

Devereaux PJ, Beattie WS, Choi PT, Badner NH, Guyatt GH, et al. (2005) How strong is the evidence for the use of perioperative beta blockers in non-cardiac surgery? Systematic review and meta-analysis of randomised controlled trials. BMJ 331: 313-21. Epub 2005 Jul 4.

Djulbegovic M, Beyth RJ, Neuberger MM, Stoffs TL, Vieweg J, et al. (2010) Screening for prostate cancer: systematic review and meta-analysis of randomised controlled trials. BMJ 341:c4543. doi: 10.1136/bmj.c4543.

Ducharme FM. (2002) Anti-leukotrienes as add-on therapy to inhaled glucocorticoids in patients with asthma: systematic review of current evidence. BMJ 324:1545.

Duley L, Henderson-Smart D, Knight M, King J. (2001) Antiplatelet drugs for prevention of pre-eclampsia and its consequences: systematic review. BMJ 322: 329-33.

Edwards AD1, Brocklehurst P, Gunn AJ, Halliday H, Juszczak E, et al. (2010) Neurological outcomes at 18 months of age after moderate hypothermia for perinatal hypoxic ischaemic encephalopathy: synthesis and meta-analysis of trial data. BMJ 340:c363. doi: 10.1136/bmj.c363.

Eikelboom JW, Quinlan DJ, Douketis JD. (2001) Extended-duration prophylaxis against venous thromboembolism after total hip or knee replacement: a meta-analysis of the randomised trials. Lancet 358: 9-15.

Eisenberg E, McNicol ED, Carr DB. (2005) Efficacy and safety of opioid agonists in the treatment of neuropathic pain of nonmalignant origin: systematic review and meta-analysis of randomized controlled trials. JAMA 293:3043-52.

El-Kadiki A, Sutton AJ. (2005) Role of multivitamins and mineral supplements in preventing infections in elderly people: systematic review and meta-analysis of randomised controlled trials. BMJ 330: 871. Epub 2005 Mar 31.

Eyding D1, Lelgemann M, Grouven U, Härter M, Kromp M, et al. (2010) Reboxetine for acute treatment of major depression: systematic review and meta-analysis of published and unpublished placebo and selective serotonin reuptake inhibitor controlled trials.BMJ 341:c4737. doi: 10.1136/bmj.c4737.

Ezekowitz JA, Armstrong PW, McAlister FA. (2003) Implantable cardioverter defibrillators in primary and secondary prevention: a systematic review of randomized, controlled trials. Ann Intern Med 138:445-52.

Ezekowitz JA, Rowe BH, Dryden DM, Hooton N, Vandermeer B, et al. (2007) Systematic review: implantable cardioverter defibrillators for adults with left ventricular systolic dysfunction. Ann Intern Med 147: 251-62.

Fan T, Wang G, Mao B, Xiong Z, Zhang Y, et al. (2008) Prophylactic administration of parenteral steroids for preventing airway complications after extubation in adults: meta-analysis of randomised placebo controlled trials. BMJ 337:a1841. doi: 10.1136/bmj.a1841.

Filippini G, Munari L, Incorvaia B, Ebers GC, Polman C, et al. (2003) Interferons in relapsing remitting multiple sclerosis: a systematic review. Lancet 361: 545-52.

Fleming C, Whitlock EP, Beil TL, Lederle FA. (2005) Screening for abdominal aortic aneurysm: a best-evidence systematic review for the U.S. Preventive Services Task Force. Ann Intern Med 142:203-11.

Ford AC, Talley NJ, Spiegel BM, Foxx-Orenstein AE, Schiller L, et al. (2008) Effect of fibre, antispasmodics, and peppermint oil in the treatment of irritable bowel syndrome: systematic review and meta-analysis. BMJ 337:a2313. doi: 10.1136/bmj.a2313.

Friedrich JO, Adhikari N, Herridge MS, Beyene J. (2005) Meta-analysis: low-dose dopamine increases urine output but does not prevent renal dysfunction or death. Ann Intern Med 142(7):510-24.

Fuccio L, Zagari RM, Eusebi LH, Laterza L, Cennamo V, et al. (2009) Meta-analysis: can Helicobacter pylori eradication treatment reduce the risk for gastric cancer? Ann Intern Med 151: 121-8.

Furukawa TA, McGuire H, Barbui C. (2002) Meta-analysis of effects and side effects of low dosage tricyclic antidepressants in depression: systematic review. BMJ 325: 991.

Gafter-Gvili A, Fraser A, Paul M, Leibovici L. (2005) Meta-analysis: antibiotic prophylaxis reduces mortality in neutropenic patients. Ann Intern Med 142: 979-95.

Geddes JR, Carney SM, Davies C, Furukawa TA, Kupfer DJ, et al. (2003) Relapse prevention with antidepressant drug treatment in depressive disorders: a systematic review. Lancet. 361: 653-61.

Gera T, Sachdev HP. (2002) Effect of iron supplementation on incidence of infectious illness in children: systematic review. BMJ 325: 1142.

Gibbs S, Harvey I, Sterling J, Stark R. (2002) Local treatments for cutaneous warts: systematic review. BMJ 325:461.

Gilligan D, Nicolson M, Smith I, Groen H, Dalesio O, et al. (2007) Preoperative chemotherapy in patients with resectable non-small cell lung cancer: results of the MRC LU22/NVALT 2/EORTC 08012 multicentre randomised trial and update of systematic review. Lancet. 369:1929-37.

Glass J, Lanctôt KL, Herrmann N, Sproule BA, Busto UE. (2005) Sedative hypnotics in older people with insomnia: meta-analysis of risks and benefits. BMJ 331: 1169. Epub 2005 Nov 11.

Gogia S, Sachdev HS. (2009) Neonatal vitamin A supplementation for prevention of mortality and morbidity in infancy: systematic review of randomised controlled trials. BMJ 338:b919. doi: 10.1136/bmj.b919.

Gonzalez R1, Zamora J, Gomez-Camarero J, Molinero LM, Bañares R, et al. (2008) Meta-analysis: Combination endoscopic and drug therapy to prevent variceal rebleeding in cirrhosis. Ann Intern Med 149:109-22.

Green JA, Kirwan JM, Tierney JF, Symonds P, Fresco L, et al. (2001) Survival and recurrence after concomitant chemotherapy and radiotherapy for cancer of the uterine cervix: a systematic review and meta-analysis. Lancet 358:781-6.

Guevara JP, Wolf FM, Grum CM, Clark NM. (2003) Effects of educational interventions for self management of asthma in children and adolescents: systematic review and meta-analysis. BMJ 326:1308-9.

Halonen J, Halonen P, Järvinen O, Taskinen P, Auvinen T, et al. (2007) Corticosteroids for the prevention of atrial fibrillation after cardiac surgery: a randomized controlled trial. JAMA 297:1562-7.

Harden A, Brunton G, Fletcher A, Oakley A. (2009) Teenage pregnancy and social disadvantage: systematic review integrating controlled trials and qualitative studies. BMJ 339: b4254. doi: 10.1136/bmj.b4254.

Häuser W, Bernardy K, Uçeyler N, Sommer C. (2009) Treatment of fibromyalgia syndrome with antidepressants: a meta-analysis. JAMA 301: 198-209. doi: 10.1001/jama.2008.944.

Hayward G, Thompson M, Heneghan C, Perera R, Del Mar C, et al. (2009) Corticosteroids for pain relief in sore throat: systematic review and meta-analysis. BMJ 339: b2976. doi: 10.1136/bmj.b2976.

Helmerhorst FM1, Perquin DA, Donker D, Keirse MJ. (2004) Perinatal outcome of singletons and twins after assisted conception: a systematic review of controlled studies. BMJ 328: 261. Epub 2004 Jan 23.

Herbison P, Hay-Smith J, Ellis G, Moore K. (2003) Effectiveness of anticholinergic drugs compared with placebo in the treatment of overactive bladder: systematic review. BMJ 326: 841-4.

Heyland DK, Novak F, Drover JW, Jain M, Su X, et al. (2001) Should immunonutrition become routine in critically ill patients? A systematic review of the evidence. JAMA 286: 944-53.

Highland KB, Strange C, Heffner JE. (2003) Long-term effects of inhaled corticosteroids on FEV1 in patients with chronic obstructive pulmonary disease. A meta-analysis. Ann Intern Med 138: 969-73.

Ho KM, Sheridan DJ. (2006) Meta-analysis of frusemide to prevent or treat acute renal failure. BMJ 333: 420. Epub 2006 Jul 21.

Hodson EM, Jones CA, Webster AC, Strippoli GF, Barclay PG, et al. (2005) Antiviral medications to prevent cytomegalovirus disease and early death in recipients of solid-organ transplants: a systematic review of randomised controlled trials. Lancet 365: 2105-15.

Hollingsworth JM, Rogers MA, Kaufman SR, Bradford TJ, Saint S, et al. (2006) Medical therapy to facilitate urinary stone passage: a meta-analysis. Lancet 368: 1171-9.

Hooper L, Bartlett C, Davey Smith G, Ebrahim S. (2002) Systematic review of long term effects of advice to reduce dietary salt in adults. BMJ 325: 628.

Hooper L, Brown TJ, Elliott R, Payne K, Roberts C, et al. (2004) The effectiveness of five strategies for the prevention of gastrointestinal toxicity induced by non-steroidal anti-inflammatory drugs: systematic review. BMJ 329: 948. Epub 2004 Oct 8.

Horvath K, Koch K, Jeitler K, Matyas E, Bender R, et al. (2010) Effects of treatment in women with gestational diabetes mellitus: systematic review and meta-analysis. BMJ 340: c1395. doi: 10.1136/bmj.c1395.

Hull RD, Pineo GF, Stein PD, Mah AF, MacIsaac SM, et al. (2001) Extended out-of-hospital low-molecular-weight heparin prophylaxis against deep venous thrombosis in patients after elective hip arthroplasty: a systematic review. Ann Intern Med 135: 858-69.

Humphrey LL, Helfand M, Chan BK, Woolf SH. (2002) Breast cancer screening: a summary of the evidence for the U.S. Preventive Services Task Force. Ann Intern Med 137:347-60.

Ismail K, Winkley K, Rabe-Hesketh S. (2004) Systematic review and meta-analysis of randomised controlled trials of psychological interventions to improve glycaemic control in patients with type 2 diabetes. Lancet 363: 1589-97.

Ives NJ, Stowe RL, Marro J, Counsell C, Macleod A, et al (2004) Monoamine oxidase type B inhibitors in early Parkinson's disease: meta-analysis of 17 randomised trials involving 3525 patients. BMJ 329: 593. Epub 2004 Aug 13.

Jackson JL, Shimeall W, Sessums L, Dezee KJ, Becher D, et al. (2010) Tricyclic antidepressants and headaches: systematic review and meta-analysis. BMJ 341:c5222. doi: 10.1136/bmj.c5222.

Jordan R, Gold L, Cummins C, Hyde C. (2002) Systematic review and meta-analysis of evidence for increasing numbers of drugs in antiretroviral combination therapy. BMJ 324: 757.

Jull A, Waters J, Arroll B. (2002) Pentoxifylline for treatment of venous leg ulcers: a systematic review. Lancet 359: 1550-4.

Jun M, Foote C, Lv J, Neal B, Patel A, et al. (2010) Effects of fibrates on cardiovascular outcomes: a systematic review and meta-analysis. Lancet 375: 1875-84. doi: 10.1016/S0140-6736(10)60656-3. Epub 2010 May 10.

Kalil AC, Levitsky J, Lyden E, Stoner J, Freifeld AG. (2005) Meta-analysis: the efficacy of strategies to prevent organ disease by cytomegalovirus in solid organ transplant recipients. Ann Intern Med 143: 870-80.

Keiser J, Utzinger J. (2008) Efficacy of current drugs against soil-transmitted helminth infections: systematic review and meta-analysis. JAMA 299: 1937-48. doi: 10.1001/jama.299.16.1937.

Kelly AM, Dwamena B, Cronin P, Bernstein SJ, Carlos RC. (2008) Meta-analysis: effectiveness of drugs for preventing contrast-induced nephropathy. Ann Intern Med. 148: 284-94.

Khazeni N, Bravata DM, Holty JE, Uyeki TM, Stave CD, et al. (2009) Systematic review: safety and efficacy of extended-duration antiviral chemoprophylaxis against pandemic and seasonal influenza. Ann Intern Med 151: 464-73. Epub 2009 Aug 3.

Kunz R, Friedrich C, Wolbers M, Mann JF. (2008) Meta-analysis: effect of monotherapy and combination therapy with inhibitors of the renin angiotensin system on proteinuria in renal disease. Ann Intern Med 148: 30-48. Epub 2007 Nov 5.

Laine L, Schoenfeld P, Fennerty MB. (2001) Therapy for Helicobacter pylori in patients with nonulcer dyspepsia. A meta-analysis of randomized, controlled trials. Ann Intern Med. 2001 134: 361-9.

Lam LL, Cameron PA, Schneider HG, Abramson MJ, Müller C, et al. (2010) Meta-analysis: effect of B-type natriuretic peptide testing on clinical outcomes in patients with acute dyspnea in the emergency setting. Ann Intern Med 153: 728-35. doi: 10.7326/0003-4819-153-11-201012070-00006.

Lee C, Gong Y, Brok J, Boxall EH, Gluud C. (2006) Effect of hepatitis B immunisation in newborn infants of mothers positive for hepatitis B surface antigen: systematic review and meta-analysis. BMJ 332: 328-36. Epub 2006 Jan 27.

Lee VC, Rhew DC, Dylan M, Badamgarav E, Braunstein GD, et al. (2004) Meta-analysis: angiotensin-receptor blockers in chronic heart failure and high-risk acute myocardial infarction. Ann Intern Med 141:693-704.

Legg L, Drummond A, Leonardi-Bee J, Gladman JR, Corr S, et al. (2007) Occupational therapy for patients with problems in personal activities of daily living after stroke: systematic review of randomised trials. BMJ 335: 922. Epub 2007 Sep 27

Legg L, Langhorne P; Outpatient Service Trialists. (2004) Rehabilitation therapy services for stroke patients living at home: systematic review of randomised trials. Lancet 363: 352-6.

León H, Shibata MC, Sivakumaran S, Dorgan M, Chatterley T, et al. (2008) Effect of fish oil on arrhythmias and mortality: systematic review.BMJ. 337: a2931. doi: 10.1136/bmj.a2931.

Li Z, Maglione M, Tu W, Mojica W, Arterburn D, et al. (2005) Meta-analysis: pharmacologic treatment of obesity. Ann Intern Med 142: 532-46.

Lightowler JV, Wedzicha JA, Elliott MW, Ram FS. (2003) Non-invasive positive pressure ventilation to treat respiratory failure resulting from exacerbations of chronic obstructive pulmonary disease: Cochrane systematic review and meta-analysis. BMJ 326: 185.

Lim E, Ali Z, Ali A, Routledge T, Edmonds L, et al. (2003) Indirect comparison meta-analysis of aspirin therapy after coronary surgery. BMJ 327:1309.

Liu H, Bravata DM, Olkin I, Friedlander A, Liu V, et al. (2008) Systematic review: the effects of growth hormone on athletic performance. Ann Intern Med 148: 747-58. Epub 2008 Mar 17.

Liu Z, Xiong T, Meads C. (2006) Clinical effectiveness of treatment with hyperbaric oxygen for neonatal hypoxic-ischaemic encephalopathy: systematic review of Chinese literature. BMJ 333: 374. Epub 2006 May 11.

Lo GH, LaValley M, McAlindon T, Felson DT. (2003) Intra-articular hyaluronic acid in treatment of knee osteoarthritis: a meta-analysis. JAMA 290: 3115-21.

Lord JM, Flight IH, Norman RJ. (2003) Metformin in polycystic ovary syndrome: systematic review and meta-analysis. BMJ 327: 951-3.

Luke TC, Kilbane EM, Jackson JL, Hoffman SL. (2006) Meta-analysis: convalescent blood products for Spanish influenza pneumonia: a future H5N1 treatment? Ann Intern Med 145: 599-609. Epub 2006 Aug 29.

Madsen MV, Gøtzsche PC, Hróbjartsson A. (2009) Acupuncture treatment for pain: systematic review of randomised clinical trials with acupuncture, placebo acupuncture, and no acupuncture groups. BMJ 338: a3115. doi: 10.1136/bmj.a3115.

Maier PC, Funk J, Schwarzer G, Antes G, Falck-Ytter YT. (2005) Treatment of ocular hypertension and open angle glaucoma: meta-analysis of randomised controlled trials. BMJ 331: 134. Epub 2005 Jul 1.

Manheimer E, Linde K, Lao L, Bouter LM, Berman BM. (2007) Meta-analysis: acupuncture for osteoarthritis of the knee.Ann Intern Med 146: 868-77.

Manheimer E, Zhang G, Udoff L, Haramati A, Langenberg P, et al. (2008) Effects of acupuncture on rates of pregnancy and live birth among women undergoing in vitro fertilisation: systematic review and meta-analysis. BMJ 336: 545-9. doi: 10.1136/bmj.39471.430451.BE. Epub 2008 Feb 7.

Martell BA, O'Connor PG, Kerns RD, Becker WC, Morales KH, et al. (2007) Systematic review: opioid treatment for chronic back pain: prevalence, efficacy, and association with addiction. Ann Intern Med 146: 116-27.

McAlister FA, Ezekowitz J, Hooton N, Vandermeer B, Spooner C, et al. (2007) Cardiac resynchronization therapy for patients with left ventricular systolic dysfunction: a systematic review. JAMA 297: 2502-14.

McAlister FA, Ezekowitz JA, Wiebe N, Rowe B, Spooner C, et al. (2004) Systematic review: cardiac resynchronization in patients with symptomatic heart failure. Ann Intern Med 141:381-90. Epub 2004 Aug 16.

McAlister FA, Lawson FM, Teo KK, Armstrong PW. (2001) Randomised trials of secondary prevention programmes in coronary heart disease: systematic review. BMJ 323: 957-62.

McAlister FA, Wiebe N, Ezekowitz JA, Leung AA, Armstrong PW. (2009) Meta-analysis: beta-blocker dose, heart rate reduction, and death in patients with heart failure. Ann Intern Med 150: 784-94.

Michael YL, Whitlock EP, Lin JS, Fu R, O'Connor EA, et al. (2010) Primary care-relevant interventions to prevent falling in older adults: a systematic evidence review for the U.S. Preventive Services Task Force. Ann Intern Med 153: 815-25. doi: 10.7326/0003-4819-153-12-201012210-00008.

Miller ER 3^rd^, Pastor-Barriuso R, Dalal D, Riemersma RA, Appel LJ, et al. (2005) Meta-analysis: high-dosage vitamin E supplementation may increase all-cause mortality. Ann Intern Med 142: 37-46. Epub 2004 Nov 10.

Miller J, Chan BK, Nelson HD. (2002) Postmenopausal estrogen replacement and risk for venous thromboembolism: a systematic review and meta-analysis for the U.S. Preventive Services Task Force. Ann Intern Med 136: 680-90.

Milne AC, Avenell A, Potter J. (2006) Meta-analysis: protein and energy supplementation in older people. Ann Intern Med 144: 37-48.

Minneci PC, Deans KJ, Banks SM, Eichacker PQ, Natanson C. (2004) Meta-analysis: the effect of steroids on survival and shock during sepsis depends on the dose. Ann Intern Med 141: 47-56.

Minns Lowe CJ, Barker KL, Dewey M, Sackley CM. (2007) Effectiveness of physiotherapy exercise after knee arthroplasty for osteoarthritis: systematic review and meta-analysis of randomised controlled trials. BMJ 335: 812. Epub 2007 Sep 20.

Moore D, Aveyard P, Connock M, Wang D, Fry-Smith A, et al. (2009) Effectiveness and safety of nicotine replacement therapy assisted reduction to stop smoking: systematic review and meta-analysis. BMJ 338: b1024. doi: 10.1136/bmj.b1024.

Nabi G, Cook J, N'Dow J, McClinton S. (2007) Outcomes of stenting after uncomplicated ureteroscopy: systematic review and meta-analysis. BMJ 334: 572. Epub 2007 Feb 20.

Nalluri SR, Chu D, Keresztes R, Zhu X, Wu S. (2008) Risk of venous thromboembolism with the angiogenesis inhibitor bevacizumab in cancer patients: a meta-analysis. JAMA 300: 2277-85. doi: 10.1001/jama.2008.656.

Nelson HD, Fu R, Griffin JC, Nygren P, Smith ME, et al. (2009) Systematic review: comparative effectiveness of medications to reduce risk for primary breast cancer. Ann Intern Med 151: 703-15, W-226-35. doi: 10.7326/0003-4819-151-10-200911170-00147.

Nelson HD, Tyne K, Naik A, Bougatsos C, Chan BK, et al. (2009) Screening for breast cancer: an update for the U.S. Preventive Services Task Force. Ann Intern Med 151: 727-37, W237-42. doi: 10.7326/0003-4819-151-10-200911170-00009.

Nelson HD, Vesco KK, Haney E, Fu R, Nedrow A, et al. (2006) Nonhormonal therapies for menopausal hot flashes: systematic review and meta-analysis. JAMA 295: 2057-71.

Nissen SE, Wolski K. (2007) Effect of rosiglitazone on the risk of myocardial infarction and death from cardiovascular causes. N Engl J Med 356: 2457-71. Epub 2007 May 21.

Norman JE, Mackenzie F, Owen P, Mactier H, Hanretty K, et al. (2009) Progesterone for the prevention of preterm birth in twin pregnancy (STOPPIT): a randomised, double-blind, placebo-controlled study and meta-analysis. Lancet 373: 2034-40. doi: 10.1016/S0140-6736(09)60947-8.

Nunes EV, Levin FR. (2004) Treatment of depression in patients with alcohol or other drug dependence: a meta-analysis. JAMA 291: 1887-96.

Nygren P, Fu R, Freeman M, Bougatsos C, Klebanoff M, Guise JM, et al.(2008) Evidence on the benefits and harms of screening and treating pregnant women who are asymptomatic for bacterial vaginosis: an update review for the U.S. Preventive Services Task Force. Ann Intern Med 148: 220-33.

Palmer SC, McGregor DO, Macaskill P, Craig JC, Elder GJ, et al. (2007) Meta-analysis: vitamin D compounds in chronic kidney disease. Ann Intern Med 147: 840-53.

Paramothayan S, Jones PW. (2002) Corticosteroid therapy in pulmonary sarcoidosis: a systematic review. JAMA. 287: 1301-7.

Parker MJ, Gillespie WJ, Gillespie LD. (2006) Effectiveness of hip protectors for preventing hip fractures in elderly people: systematic review. BMJ 332: 571-4. Epub 2006 Mar 2.

Phillips CO, Wright SM, Kern DE, Singa RM, Shepperd S, et al. (2004) Comprehensive discharge planning with postdischarge support for older patients with congestive heart failure: a meta-analysis. JAMA 291: 1358-67.

Pignone MP, Gaynes BN, Rushton JL, Burchell CM, Orleans CT, et al. (2002) Screening for depression in adults: a summary of the evidence for the U.S. Preventive Services Task Force. Ann Intern Med 136:765-76.

Polyzos NP, Polyzos IP, Zavos A, Valachis A, Mauri D, et al. (2010) Obstetric outcomes after treatment of periodontal disease during pregnancy: systematic review and meta-analysis. BMJ 341: c7017. doi: 10.1136/bmj.c7017.

Poole PJ, Black PN. (2001) Oral mucolytic drugs for exacerbations of chronic obstructive pulmonary disease: systematic review. BMJ 322: 1271-4.

Raina P, Santaguida P, Ismaila A, Patterson C, Cowan D, et al. (2008) Effectiveness of cholinesterase inhibitors and memantine for treating dementia: evidence review for a clinical practice guideline. Ann Intern Med 148: 379-97.

Ray KK, Seshasai SR, Wijesuriya S, Sivakumaran R, Nethercott S, et al. (2009) Effect of intensive control of glucose on cardiovascular outcomes and death in patients with diabetes mellitus: a meta-analysis of randomised controlled trials. Lancet. 373: 1765-72. doi: 10.1016/S0140-6736(09)60697-8.

Reichenbach S, Sterchi R, Scherer M, Trelle S, Bürgi E, et al. (2007) Meta-analysis: chondroitin for osteoarthritis of the knee or hip.Ann Intern Med 146: 580-90.

Ross JR, Saunders Y, Edmonds PM, Patel S, Broadley KE, et al. (2003) Systematic review of role of bisphosphonates on skeletal morbidity in metastatic cancer. BMJ 327: 469.

Rostom A, Dubé C, Lewin G, Tsertsvadze A, Barrowman N, et al. (2007) Nonsteroidal anti-inflammatory drugs and cyclooxygenase-2 inhibitors for primary prevention of colorectal cancer: a systematic review prepared for the U.S. Preventive Services Task Force. Ann Intern Med 146: 376-89.

Rothberg MB, Celestin C, Fiore LD, Lawler E, Cook JR. (2005) Warfarin plus aspirin after myocardial infarction or the acute coronary syndrome: meta-analysis with estimates of risk and benefit. Ann Intern Med 143:241-50.

Rucker D, Padwal R, Li SK, Curioni C, Lau DC. (2007) Long term pharmacotherapy for obesity and overweight: updated meta-analysis. BMJ 335: 1194-9. Epub 2007 Nov 15. Erratum in: BMJ. 2007 Nov 24;335(7629). doi: 10.1136/bmj.39406.519132.AD.

Salpeter SR, Buckley NS, Ormiston TM, Salpeter EE. (2006) Meta-analysis: effect of long-acting beta-agonists on severe asthma exacerbations and asthma-related deaths. Ann Intern Med 144:904-12. Epub 2006 Jun 5.

Salpeter SR, Ormiston TM, Salpeter EE. (2002) Cardioselective beta-blockers in patients with reactive airway disease: a meta-analysis. Ann Intern Med 137: 715-25.

Salpeter SR, Ormiston TM, Salpeter EE. (2004) Meta-analysis: respiratory tolerance to regular beta2-agonist use in patients with asthma. Ann Intern Med 140: 802-13.

Sattar N, Preiss D, Murray HM, Welsh P, Buckley BM, et al. (2010) Statins and risk of incident diabetes: a collaborative meta-analysis of randomised statin trials. Lancet 375: 735-42. doi: 10.1016/S0140-6736(09)61965-6. Epub 2010 Feb 16.

Schneider LS, Dagerman KS, Insel P. (2005) Risk of death with atypical antipsychotic drug treatment for dementia: meta-analysis of randomized placebo-controlled trials. JAMA 294: 1934-43.

Schürks M, Glynn RJ, Rist PM, Tzourio C, Kurth T. (2010) Effects of vitamin E on stroke subtypes: meta-analysis of randomised controlled trials. BMJ 341: c5702. doi: 10.1136/bmj.c5702.

Shah MR, Hasselblad V, Stevenson LW, Binanay C, O'Connor CM, et al. (2005) Impact of the pulmonary artery catheter in critically ill patients: meta-analysis of randomized clinical trials. JAMA 294: 1664-70.

Shamliyan TA, Kane RL, Wyman J, Wilt TJ. (2008) Systematic review: randomized, controlled trials of nonsurgical treatments for urinary incontinence in women. Ann Intern Med 148: 459-73. Epub 2008 Feb 11.

Shekelle PG, Hardy ML, Morton SC, Maglione M, Mojica WA, et al. (2003) Efficacy and safety of ephedra and ephedrine for weight loss and athletic performance: a meta-analysis. JAMA 289: 1537-45. Epub 2003 Mar 10.

Shojania KG, Ranji SR, McDonald KM, Grimshaw JM, Sundaram V, et al. (2006) Effects of quality improvement strategies for type 2 diabetes on glycemic control: a meta-regression analysis. JAMA 296: 427-40.

Shun-Shin M, Thompson M, Heneghan C, Perera R, Harnden A, et al. (2009) Neuraminidase inhibitors for treatment and prophylaxis of influenza in children: systematic review and meta-analysis of randomised controlled trials. BMJ 339:b3172. doi: 10.1136/bmj.b3172.

Sin DD1, McAlister FA, Man SF, Anthonisen NR. (2003) Contemporary management of chronic obstructive pulmonary disease: scientific review. JAMA. 290:2301-12.

Singh S, Loke YK, Furberg CD. (2007) Long-term risk of cardiovascular events with rosiglitazone: a meta-analysis. JAMA. 298:1189-95.

Strippoli GF, Craig M, Deeks JJ, Schena FP, Craig JC. (2004) Effects of angiotensin converting enzyme inhibitors and angiotensin II receptor antagonists on mortality and renal outcomes in diabetic nephropathy: systematic review. BMJ 329:828. Epub 2004 Sep 30.

Strippoli GF, Navaneethan SD, Johnson DW, Perkovic V, Pellegrini F, et al. (2008) Effects of statins in patients with chronic kidney disease: meta-analysis and meta-regression of randomised controlled trials.BMJ 2336: 645-51.

Stuck AE, Egger M, Hammer A, Minder CE, Beck JC. (2002) Home visits to prevent nursing home admission and functional decline in elderly people: systematic review and meta-regression analysis. JAMA. 87:1022-8.

Sung L, Nathan PC, Alibhai SM, Tomlinson GA, Beyene J (2007). Meta-analysis: effect of prophylactic hematopoietic colony-stimulating factors on mortality and outcomes of infection. Ann Intern Med 147:400-11.

Tang BM, Eslick GD, Nowson C, Smith C, Bensoussan A. (2007) Use of calcium or calcium in combination with vitamin D supplementation to prevent fractures and bone loss in people aged 50 years and older: a meta-analysis. Lancet 370:657-66.

Teo KK, Yusuf S, Pfeffer M, Torp-Pedersen C, Kober L, et al. (2002) Effects of long-term treatment with angiotensin-converting-enzyme inhibitors in the presence or absence of aspirin: a systematic review. Lancet. 360:1037-43.

ter Kuile FO, van Eijk AM, Filler SJ. (2007) Effect of sulfadoxine-pyrimethamine resistance on the efficacy of intermittent preventive therapy for malaria control during pregnancy: a systematic review. JAMA 297:2603-16.

Torgerson DJ, Bell-Syer SE. (2001) Hormone replacement therapy and prevention of nonvertebral fractures: a meta-analysis of randomized trials. JAMA 285]](22):2891-7.

Tramèr MR, von Elm E, Loubeyre P, Hauser C. (2006) Pharmacological prevention of serious anaphylactic reactions due to iodinated contrast media: systematic review. BMJ. 333:675. Epub 2006 Jul 31.

Trelle S, Shang A, Nartey L, Cassell JA, Low N. (2007) Improved effectivenss of partner notification for patients with sexually transmitted infections: systematic review. BMJ 334:354.

Turgeon AF, Hutton B, Fergusson DA, McIntyre L, Tinmouth AA, et al (2007) Meta-analysis: intravenous immunoglobulin in critically ill adult patients with sepsis. Ann Intern Med 146(:193-203.

Vijan S, Hayward RA; American College of Physicians. (2004) Pharmacologic lipid-lowering therapy in type 2 diabetes mellitus: background paper for the American College of Physicians. Ann Intern Med 140:650-8.

Vivekananthan DP, Penn MS, Sapp SK, Hsu A, Topol EJ. (2004) Use of antioxidant vitamins for the prevention of cardiovascular disease: meta-analysis of randomised trials. Lancet. 2003 Jun 14;361(9374):2017-23.

Walsh JM, Pignone M. (2004) Drug treatment of hyperlipidemia in women. JAMA 291: 2243-52.

Wang L, Manson JE, Song Y, Sesso HD. (2010) Systematic review: Vitamin D and calcium supplementation in prevention of cardiovascular events. Ann Intern Med 152:315-23. doi: 10.7326/0003-4819-152-5-201003020-00010.

Wang X, Qin X, Demirtas H, Li J, Mao G, et al. (2007) Efficacy of folic acid supplementation in stroke prevention: a meta-analysis. Lancet. 369:1876-82.

Weng CL, Zhao YT, Liu QH, Fu CJ, Sun F, et al. (2010) Meta-analysis: Noninvasive ventilation in acute cardiogenic pulmonary edema. Ann Intern Med 152: 590-600. doi: 10.7326/0003-4819-152-9-201005040-00009.

Whittington CJ, Kendall T, Fonagy P, Cottrell D, Cotgrove A, et al. (2004) Selective serotonin reuptake inhibitors in childhood depression: systematic review of published versus unpublished data. Lancet 63:1341-5.

Wiener RS, Wiener DC, Larson RJ. (2008) Benefits and risks of tight glucose control in critically ill adults: a meta-analysis. JAMA. 300:933-44. doi: 10.1001/jama.300.8.933.

Wilkes MM, Navickis RJ. (2001) Patient survival after human albumin administration. A meta-analysis of randomized, controlled trials. Ann Intern Med 135:149-64.

Winzenberg T, Shaw K, Fryer J, Jones G. (2006) Effects of calcium supplementation on bone density in healthy children: meta-analysis of randomised controlled trials. BMJ 333: 775. Epub 2006 Sep 15.

Wong MC, Chung JW, Wong TK. (2007) Effects of treatments for symptoms of painful diabetic neuropathy: systematic review. Effects of treatments for symptoms of painful diabetic neuropathy: systematic review. BMJ 335:87. Epub 2007 Jun 11.

Zoungas S, Ninomiya T, Huxley R, Cass A, Jardine M, et al. (2009) Systematic review: sodium bicarbonate treatment regimens for the prevention of contrast-induced nephropathy. Ann Intern Med 151:631-8. doi: 10.7326/0003-4819-151-9-200911030-00008.

From Cochrane Database Syst Rev

| Abalos E, Duley L, Steyn DW,Henderson-Smart DJ. (2007) Antihypertensive drug therapy formild tomoderate hypertension during pregnancy. Cochrane Database Syst Rev 1: CD002252. |
| --- |
| Abba K, Ramaratnam S, Ranganathan LN. (2010) Anthelmintics for people with neurocysticercosis. Cochrane Database Syst Rev 3: CD000215. |
| Abbas Z, Khan MA, Salih M, Jafri W. (2011) Interferon alpha for chronic hepatitis D. Cochrane Database Syst Rev 12: CD006002. |
| Abou-Setta AM, Houston B, Al-Inany HG, Farquhar C. (2013) Levonorgestrel-releasing intrauterine device (LNG-IUD) for symptomatic endometriosis following surgery. Cochrane Database Syst Rev 1: CD005072. |
| Afshari A, Wikkelsø A, Brok J, Møller AM, Wetterslev J. (2011) Thrombelastography (TEG) or thromboelastometry (ROTEM) to monitor haemotherapy versus usual care in patients with massive transfusion. Cochrane Database Syst Rev 3: CD007871. |
| Akolo C, Adetifa I, Shepperd S, Volmink J. (2010) Treatment of latent tuberculosis infection in HIV infected persons. Cochrane Database Syst Rev 1: CD000171. |
| AlBalawi ZH,Othman SS, AlFaleh K. (2011) Intranasal ipratropium bromide for the common cold. Cochrane Database Syst Rev 7: CD008231. |
| AlFaleh K, Anabrees J, Bassler D, Al-Kharfi T. (2011) Probiotics for prevention of necrotizing enterocolitis in preterm infants. Cochrane Database Syst Rev 3: CD005496. |
| Alhasso A, Glazener CMA, Pickard R, N’Dow JMO. (2005) Adrenergic drugs for urinary incontinence in adults. Cochrane Database Syst Rev 3: CD001842. |
| Allen SJ, Martinez EG, Gregorio GV, Dans LF. (2010) Probiotics for treating acute infectious diarrhoea. Cochrane Database Syst Rev 11: CD003048. |
| Al-Shahi Salman R. (2009) Haemostatic drug therapies for acute spontaneous intracerebral haemorrhage. Cochrane Database Syst Rev 4: CD005951. |
| Anim-Somuah M, Smyth RMD, Jones L. (2011) Epidural versus non-epidural or no analgesia in labour. Cochrane Database Syst Rev 12: CD000331. |
| Annane D, Bellissant E, Bollaert PE, Briegel J, Keh D, et al. (2004) Corticosteroids for treating severe sepsis and septic shock. Cochrane Database Syst Rev 1: CD002243. |
| Anotayanonth S, Subhedar NV, Neilson JP, Harigopal S. (2004) Betamimetics for inhibiting preterm labour. Cochrane Database Syst Rev 4: CD004352. |
| Archer J, Bower P, Gilbody S, Lovell K, Richards D, et al. (2012) Collaborative care for depression and anxiety problems. Cochrane Database Syst Rev 10: CD006525. |
| Arcidiacono PG, Calori G, Carrara S, McNicol ED, Testoni PA. (2011) Celiac plexus block for pancreatic cancer pain in adults. Cochrane Database Syst Rev 3: CD007519. |
| Arroll B, Kenealy T. (2005) Antibiotics for the common cold and acute purulent rhinitis. The Cochrane Database Syst Rev 3: CD000247. |
| Asano TK, McLeod RS. (2004) Non steroidal anti-inflammatory drugs (NSAID) and aspirin for preventing colorectal adenomas and carcinomas. Cochrane Database Syst Rev 1: CD004079. |
| Asplund K. (2002) Haemodilution for acute ischaemic stroke. Cochrane Database Syst Rev 4: CD000103. |
| Attia AM, Al-Inany HG. (2007) Gonadotrophins for idiopathic male factor subfertility. Cochrane Database Syst Rev 4: CD005071. |
| Austin N, Darlow BA, McGuire W. (2009) Prophylactic oral/topical non-absorbed antifungal agents to prevent invasive fungal infection in very low birth weight infants. Cochrane Database Syst Rev 4: CD003478. |
| Azarpazhooh A, Limeback H, Lawrence HP, Shah PS. (2011) Xylitol for preventing acute otitis media in children up to 12 years of age. Cochrane Database Syst Rev 11: CD007095. |
| Balogh R, Ouellette-Kuntz H, Bourne L, Lunsky Y, Colantonio A. (2008) Organising health care services for persons with an intellectual disability. Cochrane Database Syst Rev 4: CD007492. |
| Barclay-Goddard RE, Stevenson TJ, PoluhaW,MoffattM, Taback SP. (2004) Force platformfeedback for standing balance training after stroke. Cochrane Database Syst Rev 4: CD004129. |
| Bath PMW, Bath FJ. (2009) Prostacyclin and analogues for acute ischaemic stroke. The Cochrane Database Syst Rev 3 : CD000177. |
| Baumgart DC, MacDonald JK, Feagan B. (2008) Tacrolimus (FK506) for induction of remission in refractory ulcerative colitis. Cochrane Database Syst Rev 3: CD007216. |
| Belgamwar RB, Fenton M. (2005) Olanzapine IM or velotab for acutely disturbed/agitated people with suspected serious mental illnesses. Cochrane Database Syst Rev 2: CD003729. |
| BellolioMF, Gilmore RM, Stead LG. (2011) Insulin for glycaemic control in acute ischaemic stroke. Cochrane Database Syst Rev 9: CD005346. |
| Benchimol EI, Seow CH, Steinhart AH, Griffiths AM. (2008) Traditional corticosteroids for induction of remission in Crohn’s disease. Cochrane Database Syst Rev 2: CD006792. |
| Berenstein G, Ortiz Z. (2005) Megestrol acetate for treatment of anorexia-cachexia syndrome. Cochrane Database Syst Rev 2: CD004310. |
| Bhuta T, Henderson-Smart DJ. (1998) Elective high frequency jet ventilation versus conventional ventilation for respiratory distress syndrome in preterm infants. Cochrane Database Syst Rev 2: CD000328. |
| Birks J, Flicker L. (2003) Selegiline for Alzheimer’s disease. Cochrane Database Syst Rev 1: CD000442. |
| Bjelakovic G, Gluud LL, Nikolova D, Bjelakovic M, Nagorni A, et al. (2011) Antioxidant supplements for liver diseases. Cochrane Database Syst Rev 3: CD007749. |
| Bjelakovic G, Gluud LL, Nikolova D, Whitfield K, Wetterslev J, et al. (2011) Vitamin D supplementation for prevention of mortality in adults. Cochrane Database Syst Rev 7: CD007470. |
| Bloom JE, Rischin A, Johnston RV, Buchbinder R. (2012) Image-guided versus blind glucocorticoid injection for shoulder pain. Cochrane Database Syst Rev 8: CD009147. |
| Blumenauer BBTB, Cranney A, Burls A, Coyle D, Hochberg MC, et al. (2003) Etanercept for the treatment of rheumatoid arthritis. Cochrane Database Syst Rev 3: CD004525. |
| Boulvain M, Kelly AJ, Irion O. (2008) Intracervical prostaglandins for induction of labour. Cochrane Database Syst Rev 1: CD006971. |
| Bowen A, Lincoln N. (2007) Cognitive rehabilitation for spatial neglect following stroke. Cochrane Database Syst Rev 2: CD003586. |
| Bower P, Knowles S, Coventry PA, Rowland N. (2011) Counselling for mental health and psychosocial problems in primary care. Cochrane Database Syst Rev 9: CD001025. |
| Boyle RJ, Elremeli M, Hockenhull J, Cherry MG, Bulsara MK, et al.(2012) Venom immunotherapy for preventing allergic reactions to insect stings. Cochrane Database Syst Rev 10: CD008838. |
| Bradt J, Magee WL, Dileo C, Wheeler BL, McGilloway E. (2010) Music therapy for acquired brain injury. Cochrane Database Syst Rev 7: CD006787. |
| Brand M, Bizos D, O’Farrell PJR. (2010) Antibiotic prophylaxis for patients undergoing elective endoscopic retrograde cholangiopancreatography. Cochrane Database Syst Rev 10: CD007345. |
| Briel M, Bucher H, Boscacci R, Furrer H. (2006) Adjunctive corticosteroids for Pneumocystis jiroveci pneumonia in patients with HIV-infection. Cochrane Database Syst Rev 3: CD006150. |
| Brown SR, Baraza W. (2010) Chromoscopy versus conventional endoscopy for the detection of polyps in the colon and rectum. Cochrane Database Syst Rev 10: CD006439. |
| Browning GG, Rovers MM, Williamson I, Lous J, Burton MJ. (2010) Grommets (ventilation tubes) for hearing loss associated with otitis media with effusion in children. Cochrane Database Syst Rev 10: CD001801. |
| Brunner E, Rees K, Ward K, Burke M, Thorogood M. (2007) Dietary advice for reducing cardiovascular risk. Cochrane Database Syst Rev 4: CD002128. |
| Buppasiri P, Lumbiganon P, Thinkhamrop J, Ngamjarus C, Laopaiboon M. (2011) Calcium supplementation (other than for preventing or treating hypertension) for improving pregnancy and infant outcomes. Cochrane Database Syst Rev 10: CD007079. |
| Cahill K, Lancaster T, Green N. (2010) Stage-based interventions for smoking cessation. Cochrane Database Syst Rev 11: CD004492. |
| Cahill K, Stead LF, Lancaster T. (2012) Nicotine receptor partial agonists for smoking cessation. Cochrane Database Syst Rev 4: CD006103. |
| Calderon MA, Penagos M, Sheikh A, Canonica GW, Durham S. (2011) Sublingual immunotherapy for treating allergic conjunctivitis. Cochrane Database Syst Rev 7: CD007685. |
| Cameron MH, Lonergan E, Lee H. (2003) Transcutaneous Electrical Nerve Stimulation (TENS) for dementia. Cochrane Database Syst Rev 3: CD004032. |
| Campbell SE, Glazener CMA, Hunter KF, Cody JD, Moore KN. (2012) Conservative management for postprostatectomy urinary incontinence. CochraneDatabase of Systematic Reviews 1: CD001843. |
| Candelise L, Ciccone A. Gangliosides for acute ischaemic stroke. (2001) Cochrane Database Syst Rev 4: CD000094.. |
| Candy B, Jones L, Drake R, Leurent B, King M. (2011) Interventions for supporting informal caregivers of patients in the terminal phase of a disease. Cochrane Database Syst Rev 6: CD007617. |
| Cao P, De Rango P, Zannetti S, Giordano G, Ricci S, et al. (2000) Eversion versus conventional carotid endarterectomy for preventing stroke. Cochrane Database Syst Rev 4: CD001921. |
| Cates CJ, Cates MJ. (2012) Regular treatment with formoterol for chronic asthma: serious adverse events. Cochrane Database Syst Rev 4: CD006923. |
| CatesCJ, LassersonTJ, Jaeschke R. (2009) Regular treatmentwith formoterol and inhaled steroids for chronic asthma: serious adverse events. Cochrane Database Syst Rev 2: CD006924. |
| CepedaMS, Camargo F, Zea C, Valencia L. (2006) Tramadol for osteoarthritis. Cochrane Database Syst Rev 3: CD005522. |
| Chadwick DW, Marson AG. (2005) Zonisamide add-on for drug-resistant partial epilepsy. Cochrane Database Syst Rev 4: CD001416. |
| Chalmers R, Hollis S, Leonardi-Bee J, Griffiths CEM, Marsland A. (2006) Interventions for chronic palmoplantar pustulosis. Cochrane Database Syst Rev 1: CD001433. |
| Chambrone L, Sukekava F, Araújo MG, Pustiglioni FE, Chambrone LA, et al. (2009) Root coverage procedures for the treatment of localised recession-type defects. Cochrane Database Syst Rev 2: CD007161. |
| Chan RJ, Webster J, Marquart L. (2011) Information interventions for orienting patients and their carers to cancer care facilities. Cochrane Database Syst Rev 12: CD008273. |
| Chande N, McDonald JWD, MacDonald JK, Wang JJ. (2010) Unfractionated or low-molecular weight heparin for induction of remission in ulcerative colitis. Cochrane Database Syst Rev 10: CD006774. |
| ChenW, Gluud C. (2005) Vaccines for preventing hepatitis B in health-care workers. Cochrane Database Syst Rev 4: CD000100. |
| Cipriani A, Rendell JM, Geddes J. (2009) Olanzapine in long-term treatment for bipolar disorder. Cochrane Database Syst Rev 1: CD004367. |
| Cirocchi R, Trastulli S, Boselli C, Montedori A, Cavaliere D, et al. (2012) Radiofrequency ablation in the treatment of liver metastases from colorectal cancer. Cochrane Database Syst Rev 6: CD006317. |
| Clarkson JE, Worthington HV, Furness S, McCabe M, Khalid T, et al. (2010) Interventions for treating oral mucositis for patients with cancer receiving treatment. Cochrane Database Syst Rev 8: CD001973. |
| Conde-Agudelo A, Belizán JM,Diaz-Rossello J.(2011) Kangaroo mother care to reduce morbidity and mortality in low birthweight infants. Cochrane Database Syst Rev 3: CD002771. |
| Cook LA, Pun A, Gallo MF, Lopez LM, Van Vliet HAAM. (2007) Scalpel versus no-scalpel incision for vasectomy. Cochrane Database Syst Rev 2: CD004112. |
| Coupar F, Pollock A, van Wijck F, Morris J, Langhorne P. (2010) Simultaneous bilateral training for improving arm function after stroke. Cochrane Database Syst Rev 4: CD006432. |
| Craft AP, Finer N, Barrington KJ. (2000) Vancomycin for prophylaxis against sepsis in preterm neonates. Cochrane Database Syst Rev 1: CD001971. |
| Crosbie D, Black C, McIntyre L, Royle P, Thomas S. (2007) Dehydroepiandrosterone for systemic lupus erythematosus. Cochrane Database Syst Rev 4: CD005114. |
| Daly B, Sharif MO, Newton T, Jones K, Worthington HV. (2012) Local interventions for the management of alveolar osteitis (dry socket). Cochrane Database Syst Rev 12: CD006968. |
| Daly C, Campbell MK, Cody JD, Grant A, Khan I, et al. (2000) Double bag or Y-set versus standard transfer systems for continuous ambulatory peritoneal dialysis in end-stage renal disease. Cochrane Database Syst Rev 3: CD003078. |
| Dans AL, Tan FN, Villarruz-Sulit EC. (2002) Chelation therapy for atherosclerotic cardiovascular disease. Cochrane Database Syst Rev 4: CD002785. |
| Darlow BA, Austin N. (2003) Selenium supplementation to prevent short-term morbidity in preterm neonates. Cochrane Database Syst Rev 4: CD003312. |
| David A, Adams CE, EisenbruchM, Quraishi SN, Rathbone J.(2004) Depot fluphenazine decanoate and enanthate for schizophrenia. Cochrane Database Syst Rev 2: CD000307. |
| de Jongh T, Gurol-Urganci I, Vodopivec-Jamsek V, Car J, Atun R. (2012) Mobile phone messaging for facilitating selfmanagement of long-term illnesses. Cochrane Database Syst Rev 12: CD007459. |
| Del-Rio-Navarro BE, Espinosa-Rosales FJ, Flenady V, Sienra-Monge JJL. (2006) Immunostimulants for preventing respiratory tract infection in children. Cochrane Database Syst Rev 4: CD004974. |
| DennisCL,Hodnett ED. (2007) Psychosocial and psychological interventions for treating postpartumdepression. Cochrane Database Syst Rev 4: CD006116. |
| Derry S, Faura C, Edwards J, McQuay HJ, Moore RA. (2010) Single dose dipyrone for acute postoperative pain. Cochrane Database Syst Rev 9: CD003227. |
| Derry S, Moore RA. (2012) Topical capsaicin (low concentration) for chronic neuropathic pain in adults. Cochrane Database Syst Rev 9: CD010111. |
| DiGuiseppi C, GossCW, Higgins JPT. (2001) Interventions for promoting smoke alarmownership and function. Cochrane Database Syst Rev 2: CD002246. |
| DiNisioM, Porreca E, FerranteN, OttenHM, Cuccurullo F, et al. (2012) Primary prophylaxis for venous thromboembolism in ambulatory cancer patients receiving chemotherapy. Cochrane Database Syst Rev 2: CD008500.. |
| Dodd JM, Flenady V, Cincotta R, Crowther CA. (2006) Prenatal administration of progesterone for preventing preterm birth in women considered to be at risk of preterm birth. Cochrane Database Syst Rev 1: CD004947. |
| Donovan TJ, Buchanan K. (2012) Medications for increasing milk supply in mothers expressing breastmilk for their preterm hospitalised infants. Cochrane Database Syst Rev 3: CD005544. |
| Dowswell T, Kelly AJ, Livio S, Norman JE, Alfirevic Z. (2010) Different methods for the induction of labour in outpatient settings. Cochrane Database Syst Rev 8: CD007701. |
| Ducharme FM. (2004) Addition of anti-leukotriene agents to inhaled corticosteroids for chronic asthma. Cochrane Database Syst Rev 1: CD003133. |
| Durieux P, Trinquart L, Colombet I, Niès J, WaltonRT, et al.(2008) Computerized advice on drug dosage to improve prescribing practice. Cochrane Database Syst Rev 3: CD002894. |
| Ebrahim S, Taylor F, Ward K, Beswick A, Burke M, et al. (2011) Multiple risk factor interventions for primary prevention of coronary heart disease. Cochrane Database Syst Rev 1: CD001561. |
| Eccleston C, Palermo TM, Williams ACDC, Lewandowski A, Morley S, et al. (2012) Psychological therapies for the management of chronic and recurrent pain in children and adolescents. Cochrane Database Syst Rev 12: CD003968. |
| Edwards AGK, Hulbert-Williams N, Neal RD. (2008) Psychological interventions forwomenwithmetastatic breast cancer. Cochrane Database Syst Rev 3: CD004253. |
| Eftimov F, Winer JB, Vermeulen M, de Haan R, van Schaik IN. (2009) Intravenous immunoglobulin for chronic inflammatory demyelinating polyradiculoneuropathy. Cochrane Database Syst Rev 1: CD001797. |
| Ekeland E, Heian F, Hagen KB, Abbott JM, Nordheim L. (2004) Exercise to improve self-esteem in children and young people. Cochrane Database Syst Rev 1: CD003683. |
| Engelter S, Lyrer P. (2003) Antiplatelet therapy for preventing stroke and other vascular events after carotid endarterectomy. Cochrane Database Syst Rev 3: CD001458. |
| Enriquez A, Chu IW, Mellis C, Lin WY. (2012) Nebulised deoxyribonuclease for viral bronchiolitis in children younger than 24 months. Cochrane Database Syst Rev 11: CD008395. |
| Esposito M, Grusovin MG, Felice P, Karatzopoulos G, Worthington HV, et al. (2009) Interventions for replacing missing teeth: horizontal and vertical bone augmentation techniques for dental implant treatment. Cochrane Database Syst Rev 4: CD003607. |
| Evans DJ, Levene M, Tsakmakis M. (2007) Anticonvulsants for preventing mortality and morbidity in full term newborns with perinatal asphyxia. Cochrane Database Syst Rev 3: CD001240. |
| EzraDG, Allan BDS. (2007) Topical anaesthesia alone versus topical anaesthesiawith intracameral lidocaine for phacoemulsification. Cochrane Database Syst Rev 3: CD005276. |
| Fenton M, Rathbone J, Reilly J. (2007) Thioridazine for schizophrenia. Cochrane Database Syst Rev 3: CD001944. |
| Formoso G, Perrone E, Maltoni S, Balduzzi S, D’Amico R, et al. (2012) Short and long term effects of tibolone in postmenopausal women. Cochrane Database Syst Rev 2: CD008536. |
| Foster C, Hillsdon M, Thorogood M, Kaur A, Wedatilake T. (2005) Interventions for promoting physical activity. Cochrane Database Syst Rev 1: CD003180. |
| Foster JP, ColeMJ. (2004) Oral immunoglobulin for preventing necrotizing enterocolitis in preterm and low birth weight neonates. Cochrane Database Syst Rev 1: CD001816. |
| Foxlee R, Johansson AC, Wejfalk J, Dooley L, Del Mar CB. (2006) Topical analgesia for acute otitis media. Cochrane Database Syst Rev 3: CD005657. |
| Fraser A, Goldberg E, Acosta CJ, PaulM, Leibovici L. (2007) Vaccines for preventing typhoid fever. Cochrane Database Syst Rev 3: CD001261. |
| Freitas ERFS, Soares BGO, Cardoso JR, Atallah ÁN. (2012) Incentive spirometry for preventing pulmonary complications after coronary artery bypass graft. Cochrane Database Syst Rev 9: CD004466. |
| Furness S, Glenny AM, Worthington HV, Pavitt S, Oliver R, et al DI. (2011) Interventions for the treatment of oral cavity and oropharyngeal cancer: chemotherapy. Cochrane Database Syst Rev 4: CD006386. |
| Furukawa TA, Streiner D, Young LT, Kinoshita Y. (2001) Antidepressants plus benzodiazepines for major depression. Cochrane Database Syst Rev 3: CD001026. |
| Gabriel Sanchez R, Sanchez Gomez LM, Carmona L, Roqué i Figuls M, Bonfill Cosp X. (2005) Hormone replacement therapy for preventing cardiovascular disease in post-menopausal women. Cochrane Database Syst Rev 2: CD002229. |
| Gadomski AM, Brower M. (2010) Bronchodilators for bronchiolitis. Cochrane Database Syst Rev 12: CD001266. |
| Galandi D, Schwarzer G, Bassler D, Allgaier HP. (2002) Ursodeoxycholic acid and/or antibiotics for prevention of biliary stent occlusion. Cochrane Database Syst Rev 3: CD003043. |
| Galway K, Black A, Cantwell M, Cardwell CR, Mills M, et al. (2012) Psychosocial interventions to improve quality of life and emotional wellbeing for recently diagnosed cancer patients. Cochrane Database Syst Rev 11: CD007064. |
| Gandolfo C, Sandercock PAG, Conti M. (2002) Lubeluzole for acute ischaemic stroke. Cochrane Database Syst Rev 1: CD001924. |
| Gates S, Anderson ER. (2005) Wound drainage for caesarean section. Cochrane Database Syst Rev 1: CD004549. |
| Geraghty AJ, Welch K. (2011) Antithrombotic agents for preventing thrombosis after infrainguinal arterial bypass surgery. Cochrane Database Syst Rev 6: CD000536. |
| Gholitabar M, McGuire H, Rennie J, Manning D, Lai R. (2012) Clofibrate in combination with phototherapy for unconjugated neonatal hyperbilirubinaemia. Cochrane Database Syst Rev 12: CD009017. |
| Gibson PG, Powell H, Wilson A, Abramson MJ, Haywood P, et al. (2002) Selfmanagement education and regular practitioner review for adults with asthma. Cochrane Database Syst Rev 3: CD001117. |
| Gillies D, Taylor F, Gray C, O’Brien L, D’Abrew N. (2012) Psychological therapies for the treatment of post-traumatic stress disorder in children and adolescents. Cochrane Database Syst Rev 12: CD006726. |
| Gisbert JP, Khorrami S, Carballo F, Calvet X, Gené E, et al. (2004) Helicobacter pylori eradication therapy vs. antisecretory non-eradication therapy (with or without long-term maintenance antisecretory therapy) for the prevention of recurrent bleeding from peptic ulcer. Cochrane Database Syst Rev 2: CD004062. |
| GlazenerCMA, Evans JHC, Peto RE. (2004) Complex behavioural and educational interventions for nocturnal enuresis in children.Cochrane Database Syst Rev 1: CD004668. |
| Gluud LL, Klingenberg SL, Langholz E. (2012) Tranexamic acid for upper gastrointestinal bleeding. Cochrane Database Syst Rev 1: CD006640. |
| Gomes Jr CAR, Lustosa SAS, Matos D, Andriolo RB, Waisberg DR, et al. (2012) Percutaneous endoscopic gastrostomy versus nasogastric tube feeding for adults with swallowing disturbances. Cochrane Database Syst Rev 3: CD008096. |
| Gong Y, Gluud C. (2004) Colchicine for primary biliary cirrhosis. Cochrane Database Syst Rev 2: CD004481. |
| González U, Pinart M, Reveiz L, Alvar J. (2008) Interventions for Old World cutaneous leishmaniasis. Cochrane Database Syst Rev 4: CD005067. |
| Gordon M, Naidoo K, Akobeng AK, Thomas AG. (2012) Osmotic and stimulant laxatives for the management of childhood constipation. Cochrane Database Syst Rev 7: CD009118. |
| Gøtzsche PC, Johansen HK, Schmidt LM, Burr ML. (2004) House dust mite control measures for asthma. Cochrane Database Syst Rev 4: CD001187. |
| Gourlay SG, Stead LF, Benowitz N. (2004) Clonidine for smoking cessation. Cochrane Database Syst Rev 3: CD000058. |
| Graves PM, Gelband H. (2006) Vaccines for preventing malaria (SPf66). Cochrane Database Syst Rev 2: CD005966. |
| Green S, Buchbinder R, Barnsley L, Hall S, White M, et al. (2001) Non-steroidal anti-inflammatory drugs (NSAIDs) for treating lateral elbow pain in adults. Cochrane Database Syst Rev 4: CD003686. |
| Griffin XL, Costa ML, Parsons N, Smith N. (2011) Electromagnetic field stimulation for treating delayed union or nonunion of long bone fractures in adults. Cochrane Database Syst Rev 4: CD008471. |
| Grimes DA, Lopez LM, Schulz KF. (1999) Antibiotic prophylaxis for intrauterine contraceptive device insertion. Cochrane Database Syst Rev 3: CD001327. |
| Gurusamy KS, Kumar Y, Sharma D, Davidson BR. (2008) Ischaemic preconditioning for liver transplantation. Cochrane Database Syst Rev 1: CD006315. |
| Gurusamy KS, Samraj K, Davidson BR. (2007) Routine abdominal drainage for uncomplicated liver resection. Cochrane Database Syst Rev 3: CD006232. |
| Gurusamy KS, Samraj K, Mullerat P, Davidson BR. (2007) Routine abdominal drainage for uncomplicated laparoscopic cholecystectomy. Cochrane Database Syst Rev 4: CD006004. |
| Gurusamy KS, Samraj K. (2007) Routine abdominal drainage for uncomplicated open cholecystectomy. Cochrane Database Syst Rev 2: CD006003. |
| Haider BA, Humayun Q, Bhutta ZA. (2009) Effect of administration of antihelminthics for soil transmitted helminths during pregnancy. Cochrane Database Syst Rev 2: CD005547. |
| Hall PE, Derry S, Moore RA, McQuay HJ. (2009) Single dose oral lornoxicam for acute postoperative pain in adults. Cochrane Database Syst Rev 4: CD007441. |
| Hao Q, Lu Z, Dong BR, Huang CQ, Wu T. (2011) Probiotics for preventing acute upper respiratory tract infections. Cochrane Database Syst Rev 9: CD006895. |
| HartMG, Grant R, Garside R, Rogers G, SomervilleM, et al. (2008) Temozolomide forHigh Grade Glioma. Cochrane Database Syst Rev 4: CD007415. |
| Hawthorne K, Robles Y, Cannings-John R, Edwards AGK. (2008) Culturally appropriate health education for type 2 diabetes mellitus in ethnic minority groups. Cochrane Database Syst Rev 3: CD006424. |
| Herbert RD, de Noronha M, Kamper SJ. (2011) Stretching to prevent or reduce muscle soreness after exercise. Cochrane Database Syst Rev 7: CD004577. |
| Higgins JPT, Flicker L. (2000) Lecithin for dementia and cognitive impairment. Cochrane Database Syst Rev 4: CD001015. |
| Hirst C, Owusu-Ofori S. (2012) Prophylactic antibiotics for preventing pneumococcal infection in children with sickle cell disease. Cochrane Database Syst Rev 9: CD003427. |
| Ho JJ, Henderson-Smart DJ, Davis PG. (2002) Early versus delayed initiation of continuous distending pressure for respiratory distress syndrome in preterm infants. Cochrane Database Syst Rev 2: CD002975. |
| Ho MJ, Bellusci A, Wright JM. (2009) Blood pressure lowering efficacy of coenzyme Q10 for primary hypertension. Cochrane Database Syst Rev 4: CD007435. |
| Hodnett ED, Downe S, Walsh D. (2012) Alternative versus conventional institutional settings for birth. Cochrane Database Syst Rev 8: CD000012. |
| Hofmeyr GJ, Lawrie TA. (2012) Amnioinfusion for potential or suspected umbilical cord compression in labour. Cochrane Database Syst Rev 2012, 1: CD000013. |
| Hofmeyr GJ, Xu H. (2010) Amnioinfusion for meconium-stained liquor in labour. Cochrane Database Syst Rev 1: CD000014. |
| HofmeyrGJ, Cyna AM,Middleton P. (2004) Prophylactic intravenous preloading for regional analgesia in labour. CochraneDatabase of Systematic Reviews 4: CD000175. |
| Holland AE, Hill CJ, Jones AY, McDonald CF. Breathing exercises for chronic obstructive pulmonary disease. Cochrane Database Syst Rev 2012, 10: CD008250. |
| Hollands GJ, Hankins M, Marteau TM. (2010) Visual feedback of individuals’ medical imaging results for changing health behaviour. Cochrane Database Syst Rev 1: CD007434. |
| Hood, Jr. WB, Dans AL, Guyatt GH, Jaeschke R, McMurray JJV. (2004) Digitalis for treatment of heart failure in patients in sinus rhythm. Cochrane Database Syst Rev 2: CD002901. |
| Horvath T, Azman H, Kennedy GE, Rutherford GW. (2012) Mobile phone text messaging for promoting adherence to antiretroviral therapy in patients with HIV infection. Cochrane Database Syst Rev 3: CD009756. |
| Howlett A, Ohlsson A, Plakkal N. (2012) Inositol for respiratory distress syndrome in preterm infants. Cochrane Database Syst Rev 3: CD000366. |
| Hughes JR, Stead LF, Lancaster T. (2007) Antidepressants for smoking cessation. Cochrane Database Syst Rev 1: CD000031. |
| Hulzebos EHJ, Smit Y, Helders PPJM, vanMeeterenNLU. (2012) Preoperative physical therapy for elective cardiac surgery patients. Cochrane Database Syst Rev 11: CD010118. |
| Hunter R, Kennedy E, Song F, Gadon L, Irving CB. (2003) Risperidone versus typical antipsychotic medication for schizophrenia. Cochrane Database Syst Rev 2: CD000440. |
| Imberger G, McIlroy D, Pace NL, Wetterslev J, Brok J, et al. (2010) Positive end-expiratory pressure (PEEP) during anaesthesia for the prevention of mortality and postoperative pulmonary complications. Cochrane Database Syst Rev 9: CD007922. |
| Irving CB, Mumby-Croft R, Joy LA. (2006) Polyunsaturated fatty acid supplementation for schizophrenia. Cochrane Database Syst Rev 3: CD001257. |
| Jefferson T, Deeks JJ, Demicheli V, Rivetti D, RudinM. (2004) Amantadine and rimantadine for preventing and treating influenza A in adults. The Cochrane Database Syst Rev 3: CD001169. |
| Jefferson T, Rivetti A, Di Pietrantonj C, Demicheli V, Ferroni E. (2012) Vaccines for preventing influenza in healthy children. Cochrane Database Syst Rev 8: CD004879. |
| Johnson N, Bryant A, Miles T, Hogberg T, Cornes P. (2011) Adjuvant chemotherapy for endometrial cancer after hysterectomy. Cochrane Database Syst Rev 10: CD003175. |
| Jones A, Fay JK, Burr ML, Stone M, Hood K, et al. (2002) Inhaled corticosteroid effects on bone metabolism in asthma and mild chronic obstructive pulmonary disease. Cochrane Database Syst Rev 1: CD003537. |
| Kalish L, Snidvongs K, Sivasubramaniam R, Cope D, Harvey RJ. (2012) Topical steroids for nasal polyps. Cochrane Database Syst Rev 2012, 12: CD006549. |
| Kelly AJ, Munson C, Minden L. (2011) Nitric oxide donors for cervical ripening and induction of labour. Cochrane Database Syst Rev 6: CD006901. |
| Kenyon S, Boulvain M, Neilson JP. (2010) Antibiotics for preterm rupture of membranes. Cochrane Database Syst Rev 8: CD001058. |
| Kinnersley P, Edwards AGK, Hood K, Cadbury N, Ryan R, et al. (2007) Interventions before consultations for helping patients address their information needs. Cochrane Database Syst Rev 3: CD004565. |
| Kirthi V, Derry S, Moore RA, McQuay HJ. (2010) Aspirin with or without an antiemetic for acute migraine headaches in adults. Cochrane Database Syst Rev 4: CD008041. |
| Kleijnen J, Mackerras D. (1998) Vitamin E for intermittent claudication. Cochrane Database Syst Rev 1998, 1: CD000987. |
| Koning S, van der Sande R, Verhagen AP, van Suijlekom-Smit LWA, Morris AD, et al. (2012) Interventions for impetigo. Cochrane Database Syst Rev 1: CD003261. |
| Kösters JP, Gøtzsche PC. (2003) Regular self-examination or clinical examination for early detection of breast cancer. Cochrane Database Syst Rev 2: CD003373. |
| Krogsbøll LT, Jørgensen KJ, Grønhøj Larsen C, Gøtzsche PC. (2012) General health checks in adults for reducing morbidity and mortality from disease. Cochrane Database Syst Rev 10: CD009009. |
| Kumar S, Shelley M, Harrison C, Coles B, Wilt TJ, et al. (2006) Neo-adjuvant and adjuvant hormone therapy for localised and locally advanced prostate cancer. Cochrane Database Syst Rev 4: CD006019. |
| Lafuente-Lafuente C, Longas-Tejero MA, Bergmann JF, Belmin J. (2102) Antiarrhythmics for maintaining sinus rhythm after cardioversion of atrial fibrillation. Cochrane Database Syst Rev 5: CD005049. |
| Lafuente-Lafuente C, Melero-Bascones M. (2004) Active chest compression-decompression for cardiopulmonary resuscitation. Cochrane Database Syst Rev 4: CD002751. |
| LaMantia L, Milanese C, Mascoli N, D’Amico R, Weinstock-Guttman B. (2007) Cyclophosphamide formultiple sclerosis. Cochrane Database Syst Rev 1: CD002819. |
| Lancaster T, Stead LF. (2012) Silver acetate for smoking cessation. Cochrane Database Syst Rev 9: CD000191. |
| Larun L, Nordheim LV, Ekeland E, Hagen KB, Heian F. (2006) Exercise in prevention and treatment of anxiety and depression among children and young people. Cochrane Database Syst Rev 3: CD004691. |
| Leach JP,Marson AG,Hutton JL. (2002) Remacemide for drug-resistant localization related epilepsy. Cochrane Database Syst Rev 4: CD001900. |
| Lethaby A, Temple J, Santy J. (2008) Pin site care for preventing infections associated with external bone fixators and pins. Cochrane Database Syst Rev 4: CD004551. |
| Leucht S, Kissling W, McGrath J. (2007) Lithium for schizophrenia. Cochrane Database Syst Rev 3: CD003834. |
| Li Q, Chen N, Yang J, Zhou M, Zhou D, et al. (2009) Antiviral treatment for preventing postherpetic neuralgia. Cochrane Database Syst Rev 2: CD006866. |
| Lim J, Lasserson TJ, Fleetham J, Wright JJ. (2006) Oral appliances for obstructive sleep apnoea. Cochrane Database Syst Rev 1: CD004435. |
| Linde K, BernerMM, Kriston L. (2008) St John’s wort for major depression. Cochrane Database Syst Rev 4: CD000448. |
| Lip GYH, Wrigley BJ, Pisters R. (2012) Anticoagulation versus placebo for heart failure in sinus rhythm. Cochrane Database Syst Rev 6: CD003336. |
| Lipp A, Edwards P. (2002) Disposable surgical facemasks for preventing surgical wound infection in clean surgery. CochraneDatabase of Systematic Reviews 1: CD002929. |
| Lipp A, Lusardi G. (2006) Systemic antimicrobial prophylaxis for percutaneous endoscopic gastrostomy. Cochrane Database Syst Rev 4: CD005571. |
| Liu JP, Nikolova D, Fei Y. (2009) Immunoglobulins for preventing hepatitis A. Cochrane Database Syst Rev 2: CD004181. |
| Lonergan E, Luxenberg J. (2009) Valproate preparations for agitation in dementia. Cochrane Database Syst Rev 3: CD003945. |
| Loy C, Schneider L. (2006) Galantamine for Alzheimer’s disease and mild cognitive impairment. Cochrane Database Syst Rev 1: CD001747. |
| Lutge EE, Wiysonge CS, Knight SE, Volmink J. (2012) Material incentives and enablers in the management of tuberculosis. Cochrane Database Syst Rev 1: CD007952. |
| Magee K, Campbell SG, Moher D, Rowe BH. (2008) Heparin versus placebo for acute coronary syndromes. Cochrane Database Syst Rev 2: CD003462. |
| Marchant JM, Morris PS, Gaffney J, Chang AB. (2005) Antibiotics for prolonged moist cough in children. Cochrane Database Syst Rev 4: CD004822. |
| Marinho VCC, Higgins JPT, Logan S, Sheiham A. (2002) Fluoride gels for preventing dental caries in children and adolescents. Cochrane Database Syst Rev 1: CD002280. |
| Marshall JK, Thabane M, Steinhart AH, Newman JR, Anand A, et al. (2010) Rectal 5-aminosalicylic acid for induction of remission in ulcerative colitis. Cochrane Database Syst Rev 1: CD004115. |
| Marshall M, Crowther R, Sledge WH, Rathbone J, Soares-Weiser K. (2011) Day hospital versus admission for acute psychiatric disorders. Cochrane Database Syst Rev 12: CD004026. |
| Martí-Carvajal AJ, Karakitsiou DE, Salanti G. (2012) Human recombinant activated factor VII for upper gastrointestinal bleeding in patients with liver diseases. Cochrane Database Syst Rev 3: CD004887. |
| Massey T, Derry S, Moore RA, McQuay HJ. (2010) Topical NSAIDs for acute pain in adults. Cochrane Database Syst Rev 6: CD007402. |
| Mattick RP, Breen C, Kimber J, Davoli M.(2009) Methadone maintenance therapy versus no opioid replacement therapy for opioid dependence. Cochrane Database Syst Rev 3: CD002209. |
| Maxwell L, Singh JA. (2009) Abatacept for rheumatoid arthritis. Cochrane Database Syst Rev 4: CD007277. |
| McCormack K, Scott N, Go PM, Ross SJ, Grant A, et al. (2003) Laparoscopic techniques versus open techniques for inguinal hernia repair. Cochrane Database Syst Rev 1: CD001785. |
| McDonald JWD, Tsoulis DJ, MacDonald JK, Feagan BG. (2012) Methotrexate for induction of remission in refractory Crohn’s disease. Cochrane Database Syst Rev 12: CD003459. |
| McGee RG, Bakens A, Wiley K, Riordan SM, Webster AC. (2011) Probiotics for patients with hepatic encephalopathy. Cochrane Database Syst Rev 11: CD008716. |
| McGrath J, McDonald JWD, MacDonald JK. (2004) Transdermal nicotine for induction of remission in ulcerative colitis. Cochrane Database Syst Rev 4: CD004722. |
| McGuinness B, O’Hare J, Craig D, Bullock R, Malouf R, et al. (2010) Statins for the treatment of dementia. Cochrane Database Syst Rev 8: CD007514. |
| Mead GE, Hsieh CF, Lee R, Kutlubaev MA, Claxton A, et al. (2012) Selective serotonin reuptake inhibitors (SSRIs) for stroke recovery. Cochrane Database Syst Rev 11: CD009286. |
| Meher S, Duley L. (2006) Progesterone for preventing pre-eclampsia and its complications. Cochrane Database Syst Rev 4: CD006175. |
| Mehrholz J, Friis R, Kugler J, Twork S, Storch A, et al. (2010) Treadmill training for patients with Parkinson’s disease. Cochrane Database Syst Rev 1: CD007830. |
| MeremikwuMM, Smith HJ. (1999) Blood transfusion for treating malarial anaemia. Cochrane Database Syst Rev 4: CD001475. |
| Merry SN, Hetrick SE, Cox GR, Brudevold-Iversen T, Bir JJ, et al. (2011) Psychological and educational interventions for preventing depression in children and adolescents. Cochrane Database Syst Rev 12: CD003380. |
| Miller S, Maguire LK, Macdonald G. (2011) Home-based child development interventions for preschool children from socially disadvantaged families. Cochrane Database Syst Rev 12: CD008131. |
| Moja L, Cusi C, Sterzi R, Canepari C. (2005) Selective serotonin re-uptake inhibitors (SSRIs) for preventing migraine and tension-type headaches. Cochrane Database Syst Rev 3: CD002919. |
| Moja L, Tagliabue L, Balduzzi S, Parmelli E, Pistotti V, et al. (2012) Trastuzumab containing regimens for early breast cancer. CochraneDatabase of Systematic Reviews 4. Art.No.:CD006243. |
| Montgomery P, Dennis JA. (2003)Cognitive behavioural interventions for sleep problems in adults aged 60+. Cochrane Database Syst Rev 1: CD003161. |
| Moore ER, Anderson GC, Bergman N, Dowswell T. (2012) Early skin-to-skin contact for mothers and their healthy newborn infants. Cochrane Database Syst Rev 5: CD003519. |
| Moore RA, Straube S, Wiffen PJ, Derry S, McQuay HJ. (2009) Pregabalin for acute and chronic pain in adults. Cochrane Database Syst Rev 3: CD007076. |
| Mori R, Nardin JM, Yamamoto N, Carroli G, Weeks A. (2012) Umbilical vein injection for the routine management of third stage of labour. Cochrane Database Syst Rev 3: CD006176. |
| Mössler K, Chen X, Heldal TO, Gold C. (2011) Music therapy for people with schizophrenia and schizophrenia-like disorders. Cochrane Database Syst Rev 12: CD004025. |
| Mumtaz K, Hamid S, JafriW. (2007) Endoscopic retrograde cholangiopancreaticography with or without stenting in patients with pancreaticobiliary malignancy, prior to surgery. Cochrane Database Syst Rev 3: CD006001. |
| Myers RP, Regimbeau C, Thevenot T, Leroy V, Mathurin P, et al. (2001) Interferon for acute hepatitis C. Cochrane Database Syst Rev 4: CD000369. |
| Nair P, Milan SJ, Rowe BH. (2012) Addition of intravenous aminophylline to inhaled beta2-agonists in adults with acute asthma. Cochrane Database Syst Rev 12: CD002742. |
| Nastri CO, Gibreel A, Raine-Fenning N, Maheshwari A, Ferriani RA, et al. (2012) Endometrial injury in women undergoing assisted reproductive techniques. Cochrane Database Syst Rev 7: CD009517. |
| Navarro-Sarabia F, Ariza-Ariza R, Hernandez-Cruz B, Villanueva I. (2005) Adalimumab for treating rheumatoid arthritis. Cochrane Database Syst Rev 3: CD005113. |
| Ngo K, Kotecha D, Walters JAE, Manzano L, Palazzuoli A, et al. (2010) Erythropoiesis-stimulating agents for anaemia in chronic heart failure patients. Cochrane Database Syst Rev 1: CD007613. |
| Ni Chroinin M, Lasserson TJ, Greenstone I, Ducharme FM. (2009) Addition of long-acting beta-agonists to inhaled corticosteroids for chronic asthma in children. Cochrane Database Syst Rev 3: CD007949. |
| Nicolaï SPA, Kruidenier LM, Bendermacher BLW, Prins MH, et al. (2009) Ginkgo biloba for intermittent claudication. Cochrane Database Syst Rev 2: CD006888. |
| Norris SL, Zhang X, Avenell A, Gregg E, Schmid CH, Lau J. (2005) Long-term non-pharmacological weight loss interventions for adults with prediabetes. Cochrane Database Syst Rev 2: CD005270. |
| O’Kearney RT, Anstey K, von Sanden C, Hunt A. (2006) Behavioural and cognitive behavioural therapy for obsessive compulsive disorder in children and adolescents. Cochrane Database Syst Rev 4: CD004856. |
| O’Mathúna DP, Ashford RL. (2012) Therapeutic touch for healing acute wounds. Cochrane Database Syst Rev 6: CD002766. |
| Ooi CP, Loke SC. (2012) Sweet potato for type 2 diabetes mellitus. Cochrane Database Syst Rev 2: CD009128. |
| Oringanje C, Meremikwu MM, Eko H, Esu E, Meremikwu A, et al. (2009) Interventions for preventing unintended pregnancies among adolescents. Cochrane Database Syst Rev 4: CD005215. |
| Osborn DA, Evans NJ. (2004) Early volume expansion for prevention of morbidity and mortality in very preterm infants. Cochrane Database Syst Rev 2: CD002055. |
| Osborn DA, Sinn JKH. (2007) Probiotics in infants for prevention of allergic disease and food hypersensitivity. Cochrane Database Syst Rev 4: CD006475. |
| OsiriM, Shea B, Welch V, Suarez-AlmazorME, Strand V, et al. (2002) Leflunomide for the treatment of rheumatoid arthritis. Cochrane Database Syst Rev 3: CD002047. |
| Ostelo RWJG, Costa LOP, Maher CG, de Vet HCW, van Tulder MW. (2008) Rehabilitation after lumbar disc surgery. Cochrane Database Syst Rev 4: CD003007. |
| Owers DS, Webster AC, Strippoli GFM, Kable K, Hodson EM. (2013) Pre-emptive treatment for cytomegalovirus viraemia to prevent cytomegalovirus disease in solid organ transplant recipients. Cochrane Database Syst Rev 2: CD005133. |
| Özek E, Soll R, Schimmel MS. (2010) Partial exchange transfusion to prevent neurodevelopmental disability in infants with polycythemia. CochraneDatabase of Systematic Reviews 1: CD005089. |
| Padwal RS, Rucker D, Li SK, Curioni C, Lau DCW. (2003) Long-term pharmacotherapy for obesity and overweight. Cochrane Database Syst Rev 4: CD004094. |
| Parantainen A, Verbeek JH, Lavoie MC, Pahwa M. (2011) Blunt versus sharp suture needles for preventing percutaneous exposure incidents in surgical staff. Cochrane Database Syst Rev 11: CD009170. |
| Parker MJ, Griffiths R, Appadu B. (2002) Nerve blocks (subcostal, lateral cutaneous, femoral, triple, psoas) for hip fractures. Cochrane Database Syst Rev 1: CD001159. |
| Pepas L, Kaushik S, Bryant A, Nordin A, Dickinson HO. (2011) Medical interventions for high grade vulval intraepithelial neoplasia. Cochrane Database Syst Rev 4: CD007924. |
| Petrucci N, Iacovelli W. (2007) Lung protective ventilation strategy for the acute respiratory distress syndrome. Cochrane Database Syst Rev 3: CD003844. |
| Pidala J, Djulbegovic B, Anasetti C, Kharfan-Dabaja M, Kumar A. (2011) Allogeneic hematopoietic cell transplantation for adult acute lymphoblastic leukemia (ALL) in first complete remission. Cochrane Database Syst Rev 10: CD008818. |
| Poropat G, Giljaca V, Stimac D, Gluud C. (2010) Bile acids for liver-transplanted patients. Cochrane Database Syst Rev 3: CD005442. |
| Poustie VJ, Smyth RL, Watling RM. (1999) Oral protein calorie supplementation for children with chronic disease. Cochrane Database Syst Rev 3: CD001914. DOI: 10.1002/14651858.CD001914. |
| Prince M, Christensen E, Gluud C. (2005) Glucocorticosteroids for primary biliary cirrhosis. Cochrane Database Syst Rev 2: CD003778. |
| Proctor M, Latthe P, Farquhar C, Khan K, Johnson N. (2005) Surgical interruption of pelvic nerve pathways for primary and secondary dysmenorrhoea. Cochrane Database Syst Rev 4: CD001896. |
| Prutsky G, Domecq JP, Salazar CA, Accinelli R. (2012) Antifibrinolytic therapy to reduce haemoptysis from any cause. Cochrane Database Syst Rev 4: CD008711. |
| Pucci E, Giuliani G, Solari A, Simi S, Minozzi S, et al. (2011) Natalizumab for relapsing remitting multiple sclerosis. Cochrane Database Syst Rev 10: CD007621. |
| Pulman J, Hemming K, Marson AG. (2008) Pregabalin add-on for drug-resistant partial epilepsy. Cochrane Database Syst Rev 1: CD005612. |
| Rambaldi A, Jacobs BP, Gluud C. (2007) Milk thistle for alcoholic and/or hepatitis B or C virus liver diseases. Cochrane Database Syst Rev 4: CD003620. |
| Reda AA, Kotz D, Evers SMAA, van Schayck CP. (2012) Healthcare financing systems for increasing the use of tobacco dependence treatment. Cochrane Database Syst Rev 6: CD004305. |
| Reda S, Rowett M, Makhoul S. (2001) Prompts to encourage appointment attendance for people with serious mental illness. Cochrane Database Syst Rev 2: CD002085. |
| ReiterM, Bucek R, Stümpflen A, Minar E. (2003) Prostanoids for intermittent claudication. Cochrane Database Syst Rev 4: CD000986. |
| RenfrewMJ, McCormick FM, Wade A, Quinn B, Dowswell T. (2012) Support for healthy breastfeeding mothers with healthy term babies. Cochrane Database Syst Rev 5: CD001141. |
| Ried K, Sullivan TR, Fakler P, Frank OR, Stocks NP. (2012) ffect of cocoa on blood pressure. Cochrane Database Syst Rev 2012, 8: CD008893. |
| Riemsma RP, Kirwan JR, Taal E, Rasker HJJ. (2003) Patient education for adults with rheumatoid arthritis. Cochrane Database Syst Rev 2: CD003688. |
| Roberts L, Ahmed I, Hall S. (2007) Intercessory prayer for the alleviation of ill health. Cochrane Database Syst Rev 1: CD000368. |
| Roberts NP, Kitchiner NJ, Kenardy J, Bisson JI. (2010) Early psychological interventions to treat acute traumatic stress symptoms. Cochrane Database Syst Rev 3: CD007944. |
| Roos YB, RinkelGJE, Vermeulen M, Algra A, vanGijn J. (2003) Antifibrinolytic therapy for aneurysmal subarachnoid haemorrhage. Cochrane Database Syst Rev 2: CD001245. |
| Rosa DD, Medeiros LRF, EdelweissMI, Pohlmann PR, Stein AT. (2012) Adjuvant platinum-based chemotherapy for early stage cervical cancer. Cochrane Database Syst Rev 6: CD005342. |
| Rose SC, Bisson J, Churchill R, Wessely S. (2002) Psychological debriefing for preventing post traumatic stress disorder (PTSD). Cochrane Database Syst Rev 2: CD000560. |
| Rosti-Otajärvi EM, Hämäläinen PI. (2011) Neuropsychological rehabilitation for multiple sclerosis. Cochrane Database Syst Rev 11: CD009131. |
| Roth L, MacDonald JK, McDonald JWD, Chande N. (2011) Sargramostim (GM-CSF) for induction of remission in Crohn’s disease. Cochrane Database Syst Rev 11: CD008538. |
| Rowe BH, Bretzlaff J, Bourdon C, Bota G, Blitz S, et al. (2000) Magnesium sulfate for treating exacerbations of acute asthma in the emergency department. Cochrane Database Syst Rev 1: CD001490. |
| Rowe BH, Spooner C, Ducharme F, Bretzlaff J, BotaG. (2001) Early emergency department treatment of acute asthma with systemic corticosteroids. Cochrane Database Syst Rev 1: CD002178. |
| Rudic JS, Poropat G, Krstic MN, Bjelakovic G, Gluud C. (2012) Bezafibrate for primary biliary cirrhosis. Cochrane Database Syst Rev 1: CD009145. |
| Sahar T, Cohen MJ, Ne’eman V, Kandel L, Odebiyi DO, et al. (2007) Insoles for prevention and treatment of back pain. Cochrane Database Syst Rev 4: CD005275. |
| Sandercock PAG, Counsell C, Gubitz GJ, Tseng MC. (2008) Antiplatelet therapy for acute ischaemic stroke. Cochrane Database Syst Rev 3: CD000029. |
| Sandercock PAG, Gibson LM, Liu M. (2009) Anticoagulants for preventing recurrence following presumed non-cardioembolic ischaemic stroke or transient ischaemic attack. Cochrane Database Syst Rev 2: CD000248. |
| Sandercock PAG, Soane T. (2011) Corticosteroids for acute ischaemic stroke. Cochrane Database Syst Rev 9: CD000064. |
| Sauerland S, Jaschinski T, Neugebauer EAM. (2010) Laparoscopic versus open surgery for suspected appendicitis. Cochrane Database Syst Rev 10: CD001546. |
| Shah PS, Kaufman DA. (2009) Antistaphylococcal immunoglobulins to prevent staphylococcal infection in very low birth weight infants. Cochrane Database Syst Rev 2: CD006449. |
| Shah SS, Ohlsson A, Halliday HL, Shah VS. (2012) Inhaled versus systemic corticosteroids for the treatment of chronic lung disease in ventilated very low birth weight preterm infants. Cochrane Database Syst Rev 5: CD002057. |
| Sharma P, Blackburn RC, Parke CL, McCullough K, Marks A, Black C. (2011) Angiotensin-converting enzyme inhibitors and angiotensin receptor blockers for adults with early (stage 1 to 3) non-diabetic chronic kidney disease. Cochrane Database Syst Rev 10: CD007751. |
| Simpson TC, Needleman I, Wild SH,Moles DR, Mills EJ. (2010) Treatment of periodontal disease for glycaemic control in people with diabetes. Cochrane Database Syst Rev 5: CD004714. |
| Sinclair D, Abba K, Zaman K, Qadri F, Graves PM. (2011) Oral vaccines for preventing cholera. Cochrane Database Syst Rev 3: CD008603. |
| Singh H, Poluha W, Cheang M, Choptain N, Inegbu E, et al. (2008) Propofol for sedation during colonoscopy. Cochrane Database Syst Rev 4: CD006268. |
| Sirtori V, Corbetta D, Moja L, Gatti R. (2009) Constraint-induced movement therapy for upper extremities in stroke patients. Cochrane Database Syst Rev 4: CD004433. |
| Smedslund G, Berg RC, Hammerstrøm KT, Steiro A, Leiknes KA, et al. (2011) Motivational interviewing for substance abuse. CochraneDatabase of Systematic Reviews 5:CD008063. |
| Smith FB, Bradbury A, Fowkes G. (2012) Intravenous naftidrofuryl for critical limb ischaemia. Cochrane Database Syst Rev 7: CD002070. |
| Smith HJ, Meremikwu MM. (2003) Iron-chelating agents for treating malaria. Cochrane Database Syst Rev 2: CD001474. |
| Snidvongs K, Kalish L, Sacks R, Craig JC, Harvey RJ. (2011) Topical steroid for chronic rhinosinusitis without polyps. Cochrane Database Syst Rev 8: CD009274. |
| So PS, Jiang JY, Qin Y. (2008) Touch therapies for pain relief in adults. Cochrane Database Syst Rev 4: CD006535. . |
| Soltani H, Poulose TA, Hutchon DR. (2011) Placental cord drainage after vaginal delivery as part of the management of the third stage of labour. Cochrane Database Syst Rev 9: CD004665. |
| Sommerfield T, Price J, HiattWR. (2007) Omega-3 fatty acids for intermittent claudication. Cochrane Database Syst Rev 4: CD003833. |
| Spurling GKP, Del Mar CB, Dooley L, Foxlee R. (2007) Delayed antibiotics for respiratory infections. Cochrane Database Syst Rev 3: CD004417. |
| Steinhart AH, Ewe K, Griffiths AM, Modigliani R, Thomsen OO. (2003) Corticosteroids for maintenance of remission in Crohn’s disease. Cochrane Database Syst Rev 4: CD000301. |
| Steward DL, Grisel J, Meinzen-Derr J. (2011) Steroids for improving recovery following tonsillectomy in children. Cochrane Database Syst Rev 8: CD003997. |
| Stewart L, Burdett S, Glioma (2002) Meta-analysis Trialists Group (GMT).Chemotherapy for high-grade glioma. Cochrane Database Syst Rev 3: CD003913. |
| Straube S, Derry S, Moore RA, Wiffen PJ, McQuay HJ. (2010) Single dose oral gabapentin for established acute postoperative pain in adults. Cochrane Database Syst Rev 5: CD008183. |
| Suarez-Almazor ME, Belseck E, Shea B, Tugwell P, et al. (1998) Sulfasalazine for treating rheumatoid arthritis. Cochrane Database Syst Rev 2: CD000958. |
| Subramaniam P, Henderson-Smart DJ, Davis PG. (2005) Prophylactic nasal continuous positive airways pressure for preventing morbidity and mortality in very preterm infants. Cochrane Database Syst Rev 3: CD001243. |
| Szatmári S, Whitehouse P. (2003) Vinpocetine for cognitive impairment and dementia. Cochrane Database Syst Rev 1: CD003119.. |
| Tacklind J, MacDonald R, Rutks I, Stanke JU, Wilt TJ. (2012) Serenoa repens for benign prostatic hyperplasia. Cochrane Database Syst Rev 12: CD001423. |
| Tang H, Hunter T, Hu Y, Zhai SD, Sheng X, et al. (2012) Cabergoline for preventing ovarian hyperstimulation syndrome. Cochrane Database Syst Rev 2: CD008605. |
| Taramarcaz P, Gibson PG. (2003) Intranasal corticosteroids for asthma control in people with coexisting asthma and rhinitis. Cochrane Database Syst Rev 3: CD003570. |
| Taylor MJ, Wilder H, Bhagwagar Z, Geddes J. (2004) Inositol for depressive disorders. Cochrane Database Syst Rev 1: CD004049. |
| Thaler K, Delivuk M, Chapman A, Gaynes BN, Kaminski A, et al. (2011) Second-generation antidepressants for seasonal affective disorder. Cochrane Database Syst Rev 12: CD008591. |
| The Prophylactic Cranial Irradiation Overview Collaborative Group. (2000) Cranial irradiation for preventing brain metastases of small cell lung cancer in patients in complete remission. Cochrane Database Syst Rev 4: CD002805. |
| Theologou T, Bashir M, Rengarajan A, Khan O, Spyt T, et al. (2011) Preoperative intra aortic balloon pumps in patients undergoing coronary artery bypass grafting. Cochrane Database Syst Rev 1: CD004472. |
| Thomas LH, Cross S, Barrett J, French B, Leathley M, et al. (2008) Treatment of urinary incontinence after stroke in adults. Cochrane Database Syst Rev 1: CD004462. |
| Thomas RE, Lorenzetti D, Spragins W. (2011) Mentoring adolescents to prevent drug and alcohol use. Cochrane Database Syst Rev 11: CD007381. |
| Thompson RL, Summerbell CD, Hooper L, Higgins JPT, Little P, et al. (2003) Dietary advice given by a dietitian versus other health professional or self-help resources to reduce blood cholesterol. Cochrane Database Syst Rev 3: CD001366. |
| Thongprasom K, Carrozzo M, Furness S, Lodi G. (2011) Interventions for treating oral lichen planus. Cochrane Database Syst Rev 7: CD001168. |
| Timmer A, McDonald JWD, Tsoulis DJ, MacDonald JK. (2012) Azathioprine and 6-mercaptopurine for maintenance of remission in ulcerative colitis. Cochrane Database Syst Rev 9: CD000478. |
| Timmer A, Preiss JC, Motschall E, Rücker G, Jantschek G, et al. (2011) Psychological interventions for treatment of inflammatory bowel disease. Cochrane Database Syst Rev 2: CD006913. |
| Torvaldsen S, Roberts CL, Bell JC, Raynes-Greenow CH. (2004) Discontinuation of epidural analgesia late in labour for reducing the adverse delivery outcomes associated with epidural analgesia. Cochrane Database Syst Rev 4: CD004457. |
| Traa MX, Derry S, Moore RA. (2011) Single dose oral fenoprofen for acute postoperative pain in adults. Cochrane Database Syst Rev 2: CD007556. . |
| TristanM,Orozco LJ, Steed A, Ramírez-Morera A, Stone P. (2012) Mifepristone for uterine fibroids. Cochrane Database Syst Rev 8: CD007687. |
| Tsoi DT, Porwal M, Webster AC. (2010) Interventions for smoking cessation and reduction in individuals with schizophrenia. Cochrane Database Syst Rev 6: CD007253. |
| Tungpunkom P, Maayan N, Soares-Weiser K. (2012) Life skills programmes for chronic mental illnesses. Cochrane Database Syst Rev 1: CD000381. |
| Turner S, Arthur G, Lyons RA, Weightman AL, Mann MK, et al. (2011) Modification of the home environment for the reduction of injuries. Cochrane Database Syst Rev 2: CD003600. |
| Vale N, Nordmann AJ, Schwartz GG, de Lemos J, Colivicchi F, et al. (2011) Statins for acute coronary syndrome. Cochrane Database Syst Rev 6: CD006870. |
| van Hilten, Ramaker CC, Stowe R, Ives N. (2007) Bromocriptine/levodopa combined versus levodopa alone for early Parkinson’s disease. Cochrane Database Syst Rev 4: CD003634. |
| van Tulder MW, Touray T, Furlan AD, Solway S, Bouter LM. (2003) Muscle relaxants for non-specific low-back pain. Cochrane Database Syst Rev 4: CD004252. |
| van Wyk BE, Pillay-Van Wyk V. (2010) Preventive staff-support interventions for health workers. Cochrane Database Syst Rev 3: CD003541. |
| Venekamp RP, Thompson MJ, Hayward G, Heneghan CJ, Del Mar CB, et al. (2011) Systemic corticosteroids for acute sinusitis. Cochrane Database Syst Rev 12: CD008115. |
| Verma R, Nelson RL. (2007) Prophylactic nasogastric decompression after abdominal surgery. Cochrane Database Syst Rev 3: CD004929. |
| Verner AM, McGuire W, Craig JS. (2007) Effect of taurine supplementation on growth and development in preterm or low birth weight infants. Cochrane Database Syst Rev 4: CD006072. |
| Vidal L, Gafter-Gvili A, Leibovici L, ShpilbergO. (2009) Rituximab as maintenance therapy for patients with follicular lymphoma. Cochrane Database Syst Rev 2: CD006552. |
| Wales PW, Nasr A, de Silva N, Yamada J. (2010) Human growth hormone and glutamine for patients with short bowel syndrome. Cochrane Database Syst Rev 6: CD006321. |
| Westendorp WF, Vermeij JD, Vermeij F, Den HertogHM, Dippel DWJ, et al. (2012) Antibiotic therapy for preventing infections in patients with acute stroke. Cochrane Database Syst Rev 1: CD008530. |
| White AR, Rampes H, Liu JP, Stead LF, Campbell J. (2011) Acupuncture and related interventions for smoking cessation. Cochrane Database Syst Rev 1: CD000009. |
| Wijkstra J, Lijmer J, Balk F, Geddes J, Nolen WA. (2005) Pharmacological treatment for psychotic depression. Cochrane Database Syst Rev 4: CD004044. |
| WilkinsM, Indar A,Wormald R. (2005) Intraoperative Mitomycin C for glaucoma surgery. Cochrane Database Syst Rev 4: CD002897. |
| Wilkinson D, Ramjee G, Tholandi M, Rutherford GW. (2002) Nonoxynol-9 for preventing vaginal acquisition ofHIV infection by women from men. Cochrane Database Syst Rev 3: CD003936. |
| Williams ACDC, Eccleston C, Morley S. (2012) Psychological therapies for the management of chronic pain (excluding headache) in adults. Cochrane Database Syst Rev 11: CD007407. |
| Williams Jr JW, Aguilar C, Cornell J, Chiquette E. Dolor RJ, et al. (2003) Antibiotics for acute maxillary sinusitis. Cochrane Database Syst Rev 2: CD000243. |
| Wong PF, Chong LY, Mikhailidis DP, Robless P, Stansby G. (2011) Antiplatelet agents for intermittent claudication. Cochrane Database Syst Rev 11: CD001272. |
| Woods B, Aguirre E, Spector AE, Orrell M. (2012) Cognitive stimulation to improve cognitive functioning in people with dementia. Cochrane Database Syst Rev 2012, 2: CD005562. |
| Woolfenden S, Williams KJ, Peat J. (2001) Family and parenting interventions in children and adolescents with conduct disorder and delinquency aged 10-17. Cochrane Database Syst Rev 2: CD003015. |
| Yang W, Liu M, Teng J, Hao Z, Wu B, et al. (2011) Almitrine-Raubasine combination for dementia. Cochrane Database Syst Rev 3: CD008068. |
| Yang M, Yan Y, Yin X, Wang BY, Wu T, et al. (2010) Chest physiotherapy for pneumonia in adults. Cochrane Database Syst Rev 2: CD006338. |
| Yeung SS, Yeung EW, Gillespie LD. (2011) Interventions for preventing lower limb soft-t running injuries. Cochrane Database Syst Rev 7: CD001256. |
| Zehetner AA, Orr N, Buckmaster A, Williams K, Wheeler DM. (2010) Iron supplementation for breath-holding attacks in children. Cochrane Database Syst Rev 5: CD008132. |
| Zhu X, Proctor M, Bensoussan A, Wu E, Smith CA. (2008) Chinese herbal medicine for primary dysmenorrhoea. Cochrane Database Syst Rev 2: CD005288. |
| Zoritch B, Roberts I, Oakley A. (2000) Day care for pre-school children. Cochrane Database Syst Rev 3: CD000564. |
